# Whole genome sequencing in early onset advanced heart failure

**DOI:** 10.1038/s41598-025-88465-8

**Published:** 2025-02-05

**Authors:** Erik Linnér, Tomasz Czuba, Olof Gidlöf, Jakob Lundgren, Entela Bollano, Maria Hellberg, Selvi Celik, Neha Pimpalwar, Philipp Rentzsch, Molly Martorella, Anders Gummesson, Olle Melander, Sebastian Albinsson, Göran Dellgren, Jan Borén, Anders Jeppsson, R. Thomas Lumbers, Sonia Shah, Johan Nilsson, Pradeep Natarajan, Tuuli Lappalainen, Malin Levin, Hans Ehrencrona, J. Gustav Smith

**Affiliations:** 1 Department of Cardiology, Clinical Sciences Lund, Lund University, Lund, Sweden; 2 Department of Cardiology, Skåne University Hospital, 7 Entrégatan, 222 42 Lund, Sweden; 3 Department of Molecular and Clinical Medicine, Institute of Medicine, Gothenburg University, Gothenburg, Sweden; 4 Department of Cardiology, Sahlgrenska University Hospital, Gothenburg, Sweden; 5 Section of Clinical Genetics, Department of Clinical Genetics, Pathology and Molecular Diagnostics, Office for Medical Services, Region Skåne, Lund, Sweden; 6 Department of Gene Technology, KTH Royal Institute of Technology, Stockholm, Sweden; 7 Department of Systems Biology, Columbia University, New York, NY USA; 8 Department of Clinical Genetics and Genomics, Sahlgrenska University Hospital, Gothenburg, Sweden; 9 Department of Internal Medicine, Skåne University Hospital, Malmö, Sweden; 10 Department of Internal Medicine, Clinical Sciences Malmö, Lund University, Malmö, Sweden; 11 Section of Vascular Physiology, Department of Experimental Medical Science, Lund University, Lund, Sweden; 12 Department of Thoracic Surgery, Sahlgrenska University Hospital, Gothenburg, Sweden; 13 Institute of Health Informatics, University College London, London, UK; 14 Institute for Molecular Bioscience, University of Queensland, St Lucia, QLD Australia; 15 Department of Thoracic and Vascular Surgery, Skåne University Hospital, Lund, Sweden; 16 Thoracic Surgery and Bioinformatics Research Unit, Department of Translational Medicine, Lund University, Lund, Sweden; 17 Cardiovascular Research Center and Center for Genomic Medicine, Massachusetts General Hospital and Harvard Medical School, Boston, MA USA; 18 Program in Medical and Population Genetics and the Cardiovascular Disease Initiative (P.N.), Broad Institute of MIT and Harvard, Cambridge, MA USA; 19 Section of Clinical Genetics, Department of Laboratory Medicine, Lund University, Lund, Sweden

**Keywords:** Heart failure, Cardiomyopathies, Genetics, Genomics, Heart transplantation, Cardiology, Medical genetics

## Abstract

The genetic contributions to early onset heart failure (HF) are incompletely understood. Genetic testing in advanced HF patients undergoing heart transplantation (HTx) may yield clinical benefits, but data is limited. We performed deep-coverage whole genome sequencing (WGS) in 102 Swedish HTx recipients. Gene lists were compiled through a systematic literature review. Variants were prioritized for pathogenicity and classified manually. We also compared polygenic HF risk scores to a population-based cohort. We found a pathogenic (LP/P) variant in 34 individuals (34%). Testing yield was highest in hypertrophic (63% LP/P carriers), dilated (40%) and arrhythmogenic right ventricular (33%) cardiomyopathy and lower in ischemic cardiomyopathy (10%). A family history was more common in LP/P variant carriers than in non-carriers but was present in less than half of carriers (44% vs 13%, *P* < 0.001), whereas age was similar. Polygenic risk scores were similar in HTx recipients and the population cohort. In conclusion, we observed a high prevalence of pathogenic cardiomyopathy gene variants in individuals with early-onset advanced HF, which could not accurately be ruled out by family history and age. In contrast, we did not observe higher polygenic risk scores in early onset advanced HF cases than in the general population.

## Introduction

A genetic contribution to heart failure (HF) risk is well established^1–3^. Genetic HF risk has been shown to be more pronounced with early disease onset^2^ and to be independent of other risk factors^1,3^. Understanding the spectrum of genetic variants—genetic architecture—underlying HF risk may guide disease prediction as well as development of preventive and therapeutic strategies. Rare variants with large effect on single genes and a mendelian (monogenic) inheritance pattern have been identified since the 1990s for specific HF phenotypes including hypertrophic (HCM)^4^, arrhythmogenic right ventricular (ARVC)^5^, and dilated (DCM)^6^ cardiomyopathy. More recently, genome-wide association studies (GWAS) have demonstrated a polygenic basis affecting the risk for HF development (results from the HERMES consortium; Henry A et al., Nat Genet 2025; in press)^7^.

Current clinical practice guidelines for genetic testing are focused on cardiomyopathies and do not offer any general recommendations for testing in HF. Genetic testing is broadly recommended for HCM and ARVC but recommendations for DCM differ between guideline documents^8,9^. A subset of patients with early-onset HF progress to advanced HF, characterized by persistent symptoms despite optimal medical therapy^10^, and become eligible for heart transplantation (HTx). Current guidelines for care of HTx patients emphasize that genetic cardiomyopathies are often unrecognized but do not recommend broad genetic testing, likely due to absence of data^11^.

A few studies have investigated the yield of genetic testing in specific subpopulations of HTx recipients^12–16^, but, to our knowledge, no high-quality study has been published in the general HTx population regardless of HF phenotype.

In this study, we therefore aimed to comprehensively explore the contribution of rare and common genetic variants to early-onset advanced HF by whole genome sequencing of unselected HTx patients from the two centres in Sweden that perform heart transplantations.

## Results

### Baseline characteristics

Baseline characteristics of the early-onset advanced HF cohort are shown in Table 1 and additional characteristics as compared to the general population reference cohort MDCS are available in Supplementary Table S3. Indication for HTx was advanced heart failure—either left or right ventricle—in 101 of the patients. In four of these individuals, ventricular arrhythmias were co-indications for HTx. In one of the 102 patients, valvular prosthesis endocarditis was the HTx indication. Consequently, this patient was excluded from further analyses. Mean age at inclusion, i.e. time of HTx or bridging LVAD implantation, was 50.1 years (range 15–73) and 74% were male. Age of disease onset was not reliably available. DCM was the most common HF phenotype (n = 49, 49%), followed by ICM (n = 20, 20%), HCM including HCM phenocopies (n = 16, 16%), adult congenital heart disease (ACHD) (n = 7, 7%), ARVC (n = 6, 6%), myocarditis (n = 2, 2%) and rheumatic heart disease (n = 1, 1%). Two of the 49 patients with DCM also had hypertrabeculation indicative of left ventricular non-compaction cardiomyopathy.

**Table 1:** Baseline characteristics for the early-onset advanced heart failure cohort.

Characteristic	No. (%)							
	All (n = 101)	DCM (n = 49)	HCM (n = 16)	ARVC (n = 6)	ICM (n = 20)	ACHD (n = 7)	Myoc (n = 2)	RHD (n = 1)
Age, mean (SD), y*	50.1 (14.3)	48.1 (15.5)	53.7 (12.1)	42.0 (11.3)	60.3 (7.0)	37.9 (11.6)	34–50	59
Sex								
Female	26 (26)	12 (24)	6 (38)	4 (67)	1 (5)	0 (0)	2 (100)	1 (100)
Male	75 (74)	37 (76)	10 (63)	2 (33)	19 (95)	7 (100)	0 (0)	0 (0)
Prior genetic testing								
Yes: LP/P variant	15 (15)	4 (8)	10 (63)	1 (17)	0 (0)	0 (0)	0 (0)	0 (0)
Yes: no LP/P variant	5 (5)	1 (2)	3 (19)	1 (17)	0 (0)	0 (0)	0 (0)	0 (0)
No testing	81 (80)	44 (90)	3 (19)	4 (67)	20 (100)	7 (100)	2 (100)	1 (100)
Family history								
Yes	24 (24)	10 (20)	9 (56)	3 (50)	2 (10)	0 (0)	0 (0)	0 (0)
No	77 (76)	39 (80)	7 (44)	3 (50)	17 (90)	7 (100)	2 (100)	1 (100)

*DCM* dilated cardiomyopathy (CM), *HCM* hypertrophic CM including phenocopies, *ACM* arrhythmogenic CM, *ICM* ischemic cardiomyopathy, *ACHD* adult congenital heart disease, *Myoc*. myocarditis, *RHD* rheumatic heart disease.

*Mean (SD) for DCM, HCM, ARVC, ICM and ACHD. For myocarditis, and RHD, the range is shown instead.

### Overall yield of genetic testing for monogenic variants

Manual classification according to ACMG criteria resulted in 39 LP/P variants adjudicated to be contributing to the observed phenotype in 34 individuals (34%). Additionally, 13 VUS in 11 patients (11%) were identified as having suggestive evidence of pathogenicity consistent with exhibited phenotype. Detailed description of variant yield is available in Supplementary Results and Supplementary Table S4. When only testing genes curated by ClinGen as moderate or higher, LP/P variants were detected in 27 individuals (27%). However, in an additional four patients, HF was a part of a clinical syndrome evident even without genetic testing. Consequently, three patients had variants that would not have been detected using a restrictive approach. One of these variants were located in the dystrophin gene (*DMD)*, and the other two in the myosin light kinase 3 gene (*MYLK3)*.

### Yield of genetic testing for specific HF phenotypes

**Fig. 1:**
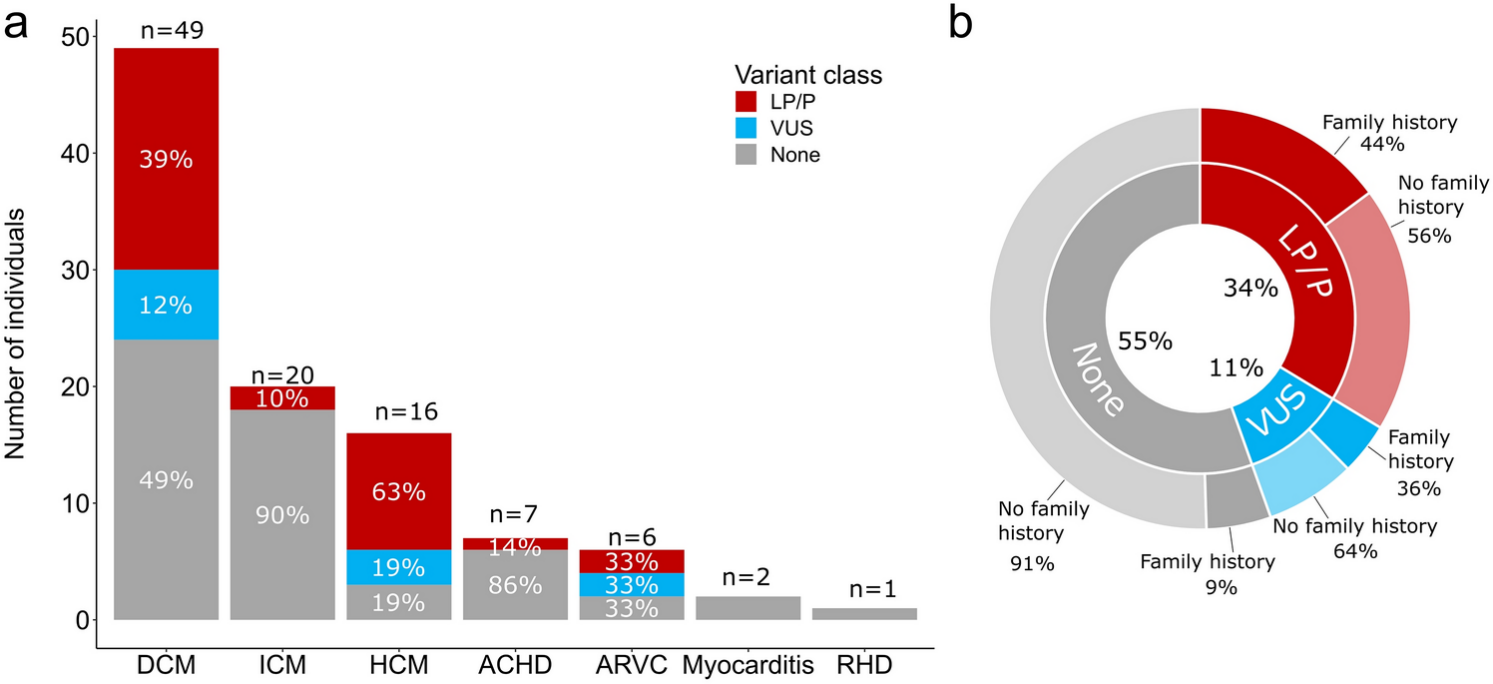
Yield of genetic testing in heart transplant recipients. Monogenic contributions to early onset advanced heart failure. (**a**) Number of individuals in which a pathogenic/likely pathogenic variant (LP/P), variant of uncertain significance with suggestive evidence of pathogenicity (VUS) or no variant of interest (none) were identified based on phenotype. (**b**) Frequency of family history based on whether a LP/P, VUS or no variant of interest was found. *DCM* dilated cardiomyopathy (CM), *ICM* ischemic CM, *HCM* hypertrophic CM including phenocopies, *ACHD* adult congenital heart disease, *ARVC* arrhythmogenic right ventricular CM, *RHD* rheumatic heart disease.

Of HF phenotypes, LP/P variants were identified most frequently in HCM (63%), followed by DCM (39%), and ARVC (33%) (Fig. 1). The largest number of LP/P variants was found in the most common phenotype DCM (19 variant carriers). No patient with myocarditis, or rheumatic heart disease carried a LP/P variant that was adjudicated to be relevant for the exhibited phenotype.

Two DCM patients with known genetic syndromes were confirmed to have pathogenic variants, a patient with glycogen storage disease XV had compound heterozygous pathogenic variants in the *GYG1* gene, and another had a variant in the *DMD* gene in a patient with Becker muscular dystrophy. Interestingly, two patients with DCM had different LP truncating variants in *MYLK3*, which only in recent studies has been associated with DCM.

Six HCM patients had known phenocopies on the basis of a genetic variant, out of which we confirmed all six: four patients with heritable transthyretin amyloidosis carried LP/P variants in *TTR*, a patient with glycogen storage disease IV was a carrier of a P variant and a rare VUS in *GBE1*, and a patient with Danon disease was a carrier of a P variant in *LAMP2*.

The yield of LP/P variants in ICM was lower (10%, 2 carriers) than for other cardiomyopathies. One patient with ICM carried a truncating *TTN* variant and another patient with ICM and polycystic kidney disease carried a LP variant in *PKD1*, a gene from the MGH panel. No other variant with evidence of pathogenicity was detected in any FH or MGH gene. Upon exclusion of ICM and ACHD, the overall yield of LP/P variants in patients with cardiomyopathy was 42% while the yield of suggestive VUS variants was 15%.

**Fig. 2:**
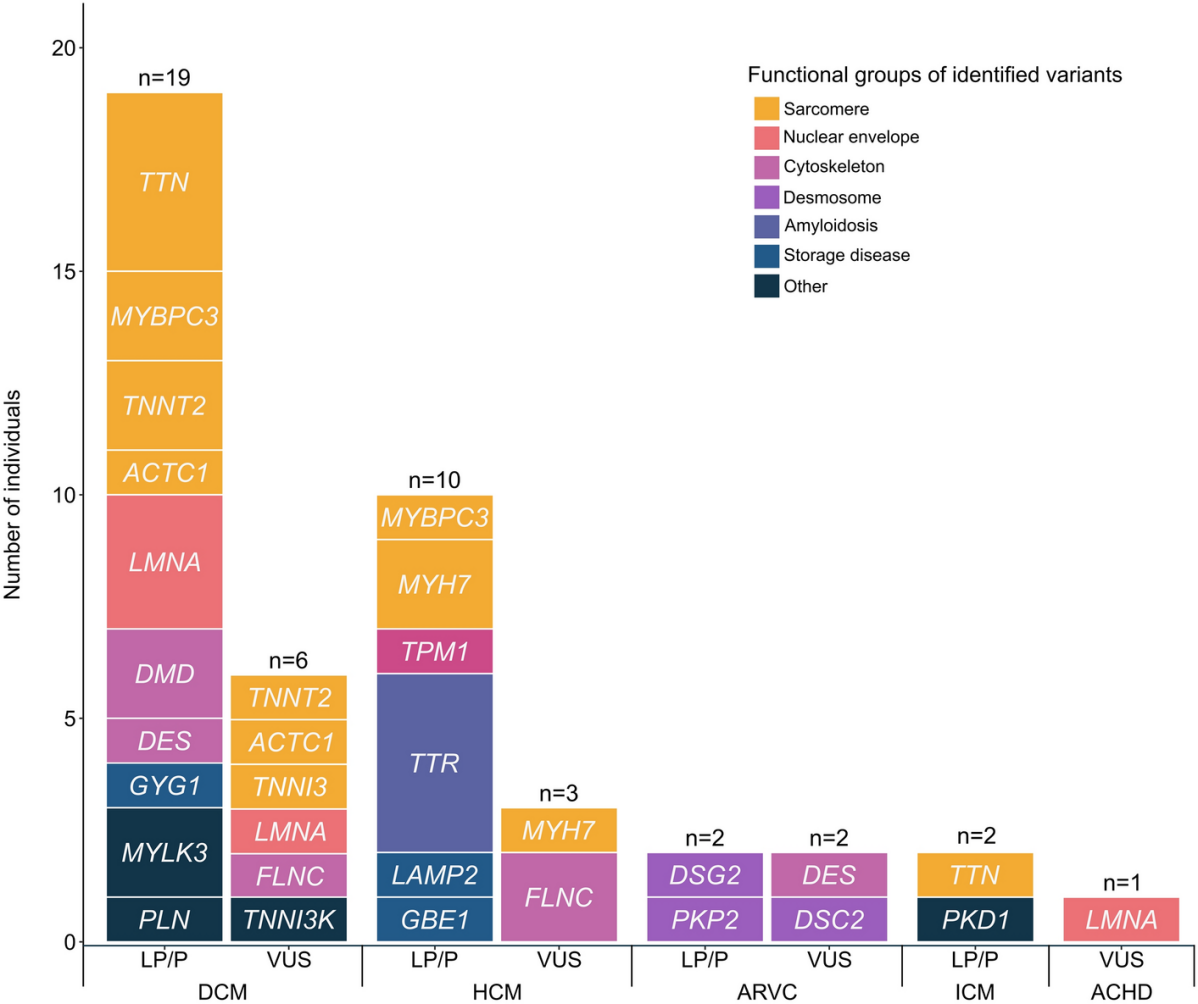
Genes identified from genetic testing of heart transplant recipients. Pathogenic variants in sarcomeric genes were most common in DCM and HCM phenotypes, while variants in desmosomal genes were mainly detected in ARVC. Storage diseases include glycogen storage disease and lysosomal storage disease. All patients with genetic amyloidosis had transthyretin amyloidosis. *DCM* dilated cardiomyopathy (CM), *HCM* hypertrophic CM including HCM phenocopies, *ARVC* arrhythmogenic right ventricular CM, *ICM* ischemic cardiomyopathy, *ACHD* adult congenital heart disease.

Most of the identified LP/P variants were located in the most well-established cardiomyopathy genes, including sarcomeric genes (n = 14, in *TTN*, *MYBPC3*, *MYH7*, *TPM1*, *TNNT2*, *ACTC1*) nuclear envelope genes (n = 3, in *LMNA*) and desmosomal genes (n = 2, in *DSG2*, *PKP2*) as shown in Fig. 2. Most identified genes and variants were concordant with the expected clinical phenotype, with a few notable exceptions. As mentioned, one ICM patient had a LP variant in *TTN*, consistent with genetic susceptibility to myocardial dysfunction to which ischemic heart disease further contributed. In two individuals, two different variants curated as pathogenic (for LQTS) was identified in *KCNQ1*, but was of unclear clinical relevance to the HF phenotype (HCM and lymphocytic myocarditis, respectively) and so were viewed as secondary findings.

### Differences by pathogenic variant carrier status

Age was similar between the patients with LP/P, VUS, and those without any significant variant (mean age 50.1, 50.0, and 50.2 years, respectively, *P* = 0.98) as shown in Table 2. A family history of cardiomyopathy was more common in LP/P carriers (44%, *P* < 0.001) compared to VUS carriers (36%) and patients without any significant variant (9%) as shown in Fig. 1. LP/P variants were identified more frequently in those with a family history than in those without (63% and 25%, *P* < 0.001).

**Table 2:** Characteristics of the early onset advanced heart failure cohort by variant carrier status.

Characteristic	No. (%)			
	Total n = 101 (100)	LP/P n = 34 (34)	VUS n = 11 (11)	None n = 56 (55)
Age, mean (SD), y	50.1 (14.3)	50.1 (14.5)	50.0 (13.2)	50.2 (14.6)
Sex				
Female	26 (26)	10 (29)	5 (45)	11 (20)
Male	75 (74)	24 (71)	6 (55)	45 (80)
Family history				
Yes	24 (24)	19 (56)	4 (36)	5 (9)
No	77 (76)	15 (44)	7 (64)	51 (91)

*LP/P* pathogenic or likely pathogenic, *VUS* variant of uncertain significance, *None* no variant of significance.

### Sensitivity analysis using a restrictive panel

In a sensitivity analysis, we evaluated a more restrictive gene panel (Supplementary Table S2) for non-syndromic heart failure cases, and additional targeted genes for syndromic cases, and identified 23 non-syndromic and 8 syndromic patients with a LP/P variant. The eight syndromic patients were: one patient with glycogen storage disease XV (*GYG1*); one patient with Becker muscular dystrophy (*DMD*); one patient with polycystic kidney disease (*PKD*); one patient with glycogen storage disease IV (*GBE1*) and four patients with systemic manifestations of transthyretin amyloidosis (*TTR*). Thus 31 of the 34 individuals would have received their molecular diagnosis if using this more restrictive approach. The three patients that would not have been detected using this approach were: one patient with DCM without any documentation of myopathy with a LP variant in *DMD*, and two patients with DCM and LP variants in *MYLK3*.

### Polygenic risk score

**Fig. 3:**
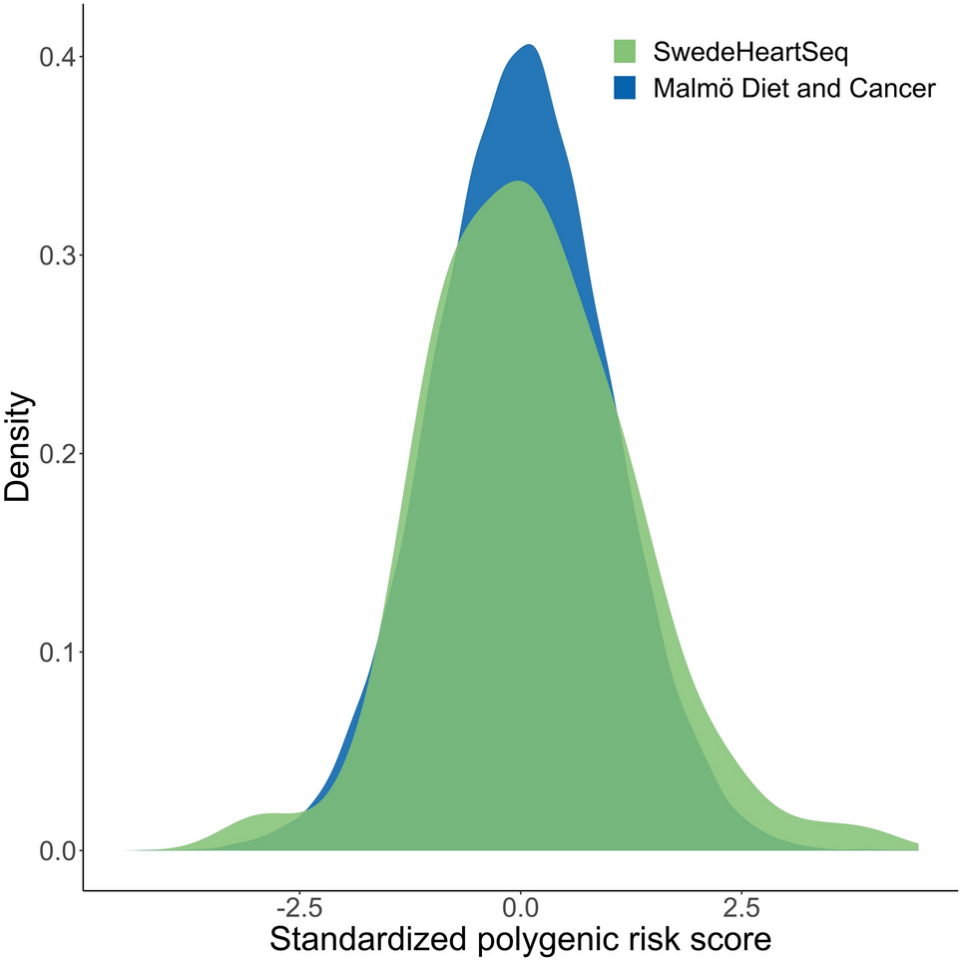
Distribution of polygenic risk score in heart transplant recipients and the general population. Polygenic risk scores are standardized to the corresponding scores of the population-based Malmö Diet and Cancer cohort.

We detected no significant difference in PRS between the HF cohort and the MDCS cohort (standardized mean polygenic risk score difference 0.10 [95% CI, − 0.10 to 0.30]), although a suggested excess of participants with high PRS (skewness 0.34 and − 0.00 in the HF and MDCS cohorts, respectively) was seen in the HF cohort as shown in Fig. 3. Kurtosis was similar in both groups, 3.8 in the HF cohort and 3.2 in the MDCS cohort. The Kolmogorov–Smirnov test did not indicate a difference in PRS distribution between the two cohorts (*P* = 0.65). Of the 101 patients, 28 individuals (28%) had a PRS higher than the 75th percentile of the MDCS cohort and 8 individuals (8%) had a PRS higher than the 95th percentile. There was no difference in PRS between individuals without and with an identified LP/P variant (0.11 and 0.081, difference 0.028 [95% CI, − 0.46 to 0.52]), Supplementary Fig. S2.

The PRS distribution of the carriers of LP/P variants had a slight positive skewness compared to the non-carriers (0.93 and 0.13). Kurtosis was similar between these groups, 4.2 and 3.7 for variant carriers and non-carriers, respectively. Kolmogorov–Smirnov test was non-significant (*P* = 0.23). There was no difference in polygenic risk scores between phenotypes (*P* = 0.86).

## Discussion

**Fig. 4:**
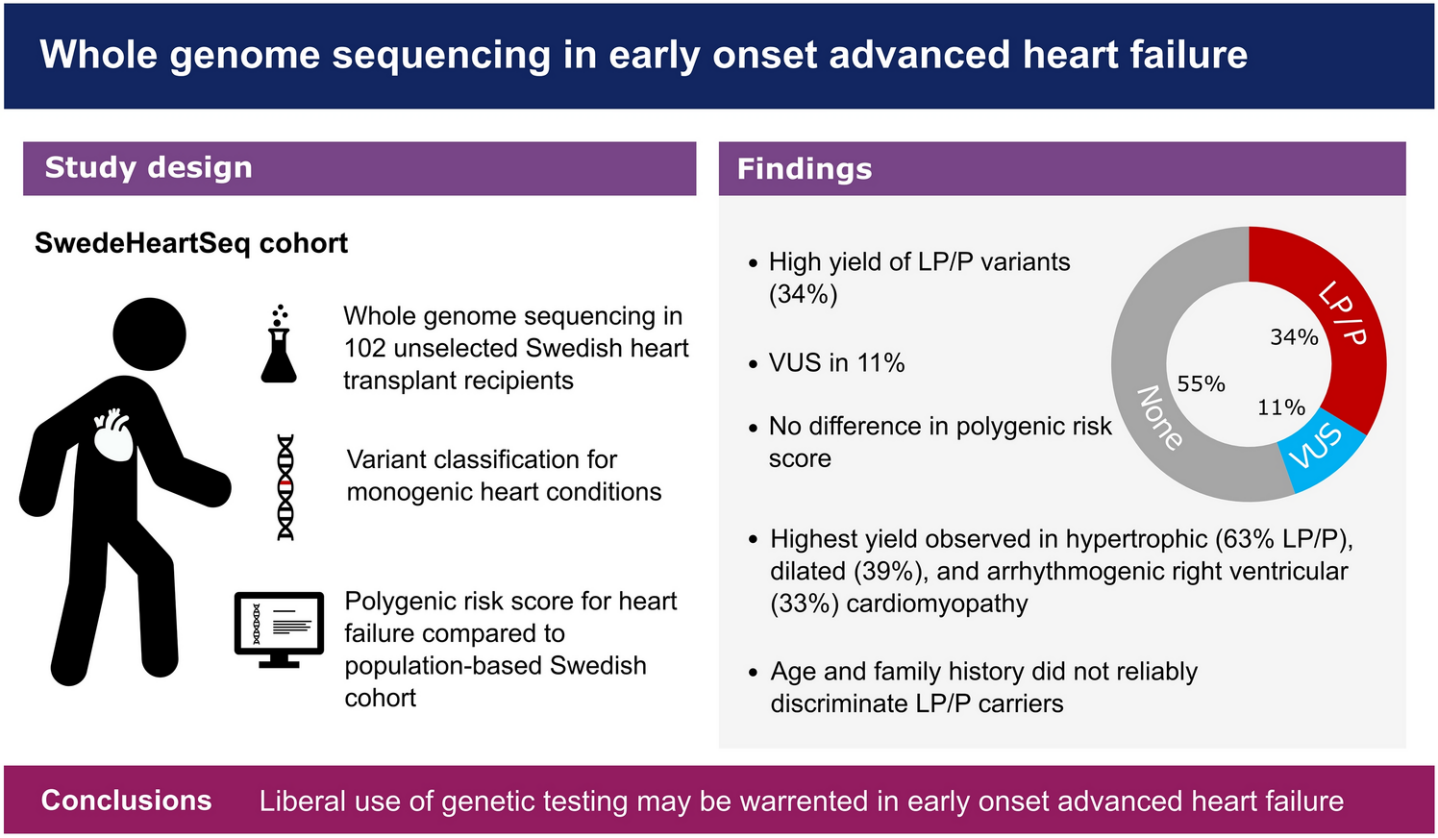
Graphical abstract. Yield of whole genome sequencing in patients with early onset advanced heart failure. *LP/P* likely pathogenic or pathogenic variant, *VUS* variant of uncertain significance.

To our knowledge, the current study represents the first study to systematically investigate the genetic architecture of early-onset advanced HF. We performed WGS in a nation-wide cohort of unselected HTx and LVAD recipients, with a mean age of 50 years at inclusion during surgery and identified a LP/P variant in 34% and a suspected pathogenic VUS variant in another 11%. In addition, all patients except one had several VUS in which pathogenicity could neither be confidently ruled in nor ruled out. The yield of genetic testing was particularly high in HCM, DCM, and ARVC but low in ICM and ACHD. No pathogenic variant in a FH gene was detected and only one pathogenic variant in a MGH gene, in a patient with polycystic kidney disease which was evident even without genetic testing. Polygenic risk scores for all-cause HF were not higher in HTx recipients compared to a reference cohort from the same population (Fig. 4).

Our findings provide insights into the genetic architecture of early-onset advanced HF. The contribution of monogenic hypertension or familial hypercholesterolemia to this HF phenotype seems limited. This observation may reflect either the later onset of HF with these conditions, the frequent involvement of other vascular territories which may result in lower priority for HTx, or that these patients have identified earlier before onset of coronary artery disease. We also observed a low yield in ACHD patients, in which germline genetic variation may play a more limited role^17^. In contrast, the prevalence of high-confidence pathogenic variants in cardiomyopathy genes was 34% and increased to 42% when excluding ICM and ACHD. This latter estimate is in line with recent studies in selected HTx recipients with non-ischemic cardiomyopathy. Specifically, Boen et al.^12^ and Kim et al.^16^ reported similarly high proportions of LP/P carriers (39% and 44%, respectively) from genetic testing of idiopathic cardiomyopathy patients undergoing HTx.

Although we identified 325 genes (Supplementary Fig. S1) to have been associated with cardiomyopathy over the three decades since the first cardiomyopathy gene (*MYH7*) was cloned in 1990^18^, most identified LP/P variants in the current study—as in most studies of genetic testing in cardiomyopathies^4–6,12–15^—were located in a small core set of mainly sarcomeric genes. These genes are central in the pathophysiology of HF and have the most robust evidence of pathogenicity. Our findings thus largely support the current trend towards more focused gene panels in cardiomyopathy testing^9^. One exception from this were two DCM patients with LP variants in *MYLK3*, which is not an established cardiomyopathy gene but has emerging evidence of pathogenicity^19,20^.

While rare pathogenic variants appear to be important for early-onset advanced HF, our findings suggest that common variants associated with HF in the population, which generally has a late onset, have limited impact on risk for this condition. The risk score overlaps significantly with scores for hypertension and coronary heart disease which are of limited importance for our phenotype, and it is therefore not unexpected that rare genetic factors of larger effect contribute more in this context.

Our findings also have implications for genetic testing. Only 20% of patients had been tested prior to our study, and low genetic testing rates in HTx recipients have also been noted in other studies^12,21^. With our strategy of broad genetic testing, the detection of LP/P variants increased to 34%, as well as a VUS with suspected pathogenicity in another 11%. Our results also suggest that genetic testing appears to result in higher yield in early-onset, advanced HF patients as compared to all-comer cardiomyopathies. This conclusion is corroborated by targeted studies in patients with DCM and HCM^13,14^. Verdonschot and coworkers demonstrated a yield of 19% in patients with DCM, ranging from 13% in non-familial DCM to 36% in familial DCM^6^. In our cohort, the overall yield in DCM was 39%, and ranged from 31% in non-familial DCM to 70% in familial DCM, demonstrating that gene testing in advanced DCM produces a high yield regardless of family history. Collectively, these findings support broad genetic testing in HTx recipients regardless of family history. The value of such testing extends mainly to family members, who can be stratified for surveillance or confident discharge, but also in some cases to the patient^22^.

Our study has several strengths, including the larger size than previous studies^12^ and broadly representative design with nation-wide all-comers. Furthermore, our use of WGS with a comprehensive gene list and detailed prioritization algorithm, with which we have substantial experience from clinical practice in Sweden, that incorporates a wide range of population cohorts and in silico prediction tools reduce the risk of undetected LP/P variants. However, our study also has limitations. First, the limited sample size results in imprecision of the yield estimates and lack of insight for less common phenotypes such as myocarditis, and rheumatic heart disease. Second, structural variants were not examined in the current study which could increase the diagnostic yield further. Mitochondrial variants were also not evaluated but are unlikely to add substantially do the yield of testing in this patient category. Third, we lacked the precise information on disease onset and used the surrogate marker of age of HTx or VAD implantation. Although severe cases are expected to progress more rapidly, some patients will have had more indolent diseases with earlier onset than expected. Finally, although polygenic risk scores for HF in the total population were not higher in early-onset advanced HF, such scores developed in sufficiently powered cohorts for early-onset HF or individual cardiomyopathy phenotypes were not available and will be needed to further understand the contribution of polygenic effects to early-onset HF.

In conclusion, we report a high burden of pathogenic variants in cardiomyopathy genes in heart transplant recipients regardless of family history. In contrast, the prevalence of pathogenic variants in MGH or FH genes was low and no enrichment of polygenic risk scores for HF was observed compared to the general population. To our knowledge, this is the first study to perform whole genome sequencing in heart transplant recipients regardless of heart failure phenotype. Our findings indicate that genetic testing for rare pathogenic cardiomyopathy variants may be considered in heart transplant recipients, particularly when presenting with HCM, DCM, or ARVC.

## Methods

### Study cohorts

All HTx procedures in Sweden, which has a population of more than 10 million people, are performed at Sahlgrenska University Hospital in Gothenburg or Skåne University Hospital in Lund. Patient selection and outcomes from HTx in Sweden has been reported to be comparable with other large institutions internationally, although recipient age is lower than at some international institutions with essentially no recipients aged older than 65 years at transplantation^23^. Biopsies from explanted hearts at both centres are routinely saved in a biobank for research and, as part of the ongoing SwedeHeartSeq research program, such samples undergo genome and transcriptome sequencing. The current report represents the first analysis from this program, which includes left ventricular apex or free wall biopsies from 102 randomly selected adults who underwent a first HTx or LVAD implantation as bridge to transplant at either of these two institutions between March 2012 and January 2021, regardless of phenotype and family history. Clinical data were collected from the electronic health records (EHR) at each hospital. HF phenotypes were determined according to guidelines from the European Society of Cardiology (ESC)^10^.

As reference cohort for the polygenic risk score and exome variants, we used a population-based cohort from southern Sweden, the Malmö Diet and Cancer study (MDCS). MDCS includes 30,447 participants randomly selected from the general population in Sweden, and has been described in detail previously^24^. Additional details regarding recruitment, genotyping, sequencing, and quality control for the MDCS are included in the Supplementary Methods. Genotypes for calculation of the polygenic risk score were available in 25,295 participants.

This study was approved by the Ethics Review Boards at Lund University and Gothenburg University. All participants provided written informed consent.

### Whole genome sequencing and data processing

DNA extraction, purification, whole genome sequencing (WGS), and data processing are detailed in the Supplementary Methods. Briefly, genomic DNA was extracted and purified from explanted myocardial biopsies. Sequencing was performed to an average target sequencing depth of 60× using paired-end 150 bp sequencing. Median coverage was 71× and coverage exceeded 30× at 90% of sites on average. Variants located in low-complexity regions were excluded^25^. Minimum quality thresholds were a mean coverage of at least 30× and variant call rate no less than 99%, for which all samples qualified.

### Heart failure gene collation

We aimed to compile an exhaustive list of genes to provide an upper bound for what can be achieved by sequencing the entire genome in HTx recipients. We included genes related to conditions that could contribute to early-onset HF including cardiomyopathies, familial hypercholesterolemia (FH) and monogenic hypertension (MGH). To this end, we compiled lists of genes curated by ClinGen^26^ and PanelApp^27^ and performed a systematic review of the literature. The process is described in detail in Supplementary Methods. Altogether, we identified 105 articles from which we abstracted 369 genes which are listed in the Supplementary Fig. S1 with references in Supplementary Table S1 including 325 genes for cardiomyopathies, 5 for FH, and 39 for MGH. As a sensitivity analysis, we also utilized a more restrictive approach using only genes curated by ClinGen for comparison (see Supplementary Methods and Supplementary Table S2 for details) with additional targeted genes in syndromic cases.

### Pathogenic variant annotation, prioritization, and classification

For variant annotation and prioritization, we developed a bioinformatic pipeline based on the pipeline used in Swedish clinical genomics departments. Variants were prioritized based on the output of annotation tools using a slightly modified version of an established variant ranking algorithm^28^ based on predicted pathogenicity. Briefly, this algorithm results in a score that incorporates in silico prediction tools, allele frequency, as well as ClinVar annotations (https://www.ncbi.nlm.nih.gov/clinvar/). A detailed description of the score is included in the Supplementary Methods. Variants were then manually classified according to the American College of Medical Genetics (ACMG) criteria^29^ and participants were divided into three groups depending on carrier status for identified variants: (1) LP/P: presence of at least one likely pathogenic (LP) or pathogenic (P) variant adjudicated to be causal or contributing to HF phenotype; (2) VUS: presence of variant of uncertain significance (VUS) with suggestive evidence of pathogenicity where additional evidence is needed; and (3) None: no variant of significance (neither LP/P variant nor VUS with suggestive evidence) was identified. Additional information on variant classification is available in the Supplementary Methods.

### Polygenic risk score

A genome-wide polygenic risk score for all-cause HF was developed comprising 1,008,426 single nucleotide variants (Shah S, PhD; unpublished data). Variant selection and weights were based on beta estimates and standard errors from tested SNPs in a genome-wide association study conducted in 153,174 HF cases and 1,793,175 controls from the HERMES consortium (Henry A et al., Nat Genet 2024; in press)^7^. A polygenic score which best captures the cumulative effect across all tested variants was developed using Bayesian regression methods in the LDpred2 algorithm^30^. Additional details on the derivation of the score are provided in the Supplementary Methods. The risk score was trimmed to include variants available in both the MDCS and HTx cohort and included > 99% of variants in the risk score (1,002,369 variants).

### Statistical analysis

Pearsons’s Chi-squared test and Fisher’s exact test were used to test for differences between categorical variables. For testing differences in means, Student’s *t*-test was used. For comparison of the difference between multiple variables, analysis of variance (ANOVA) with Bonferroni correction was used for post-hoc analyses. The polygenic scores were standardized to the mean scores and standard deviations of MDCS, and we investigated any score difference between the HTx patients and the MDCS cohort. The Kolgomorov–Smirnov test was used to test for differences between distribution in polygenic risk scores between the two cohorts.

Analyses were performed using R version 4.2.2 (The R foundation).

## Supplementary Information

Supplementary Information.

## Data Availability

The datasets generated or analysed during the current study are available through the following repositories: HERMES: the Cardiovascular Disease Knowledge Portal (https://cvd.hugeamp.org/datasets.html), Malmö Diet and Cancer Study: https://www.malmo-kohorter.lu.se/malmo-diet-cancer-mdc, SwedeHeartSeq: https://portal.research.lu.se/en/organisations/molecular-epidemiology-and-cardiology/projects/.
