# Supplementary material for: Whole genome sequencing in early onset advanced heart failure
Source: Sci Rep. 2025 Feb 5;15:4306. doi: 10.1038/s41598-025-88465-8 (PMC11799378; doi:10.1038/s41598-025-88465-8)

**Whole genome sequencing in early onset advanced heart failure**

Erik Linnér, MD; Tomasz Czuba, MSc; Olof Gidlöf, PhD; Jakob Lundgren, MD, PhD; Entela Bollano, MD, PhD; Maria Hellberg, MD; Selvi Celik, PhD; Neha Pimpalwar, PhD; Philipp Rentzsch, PhD; Molly Martorella, PhD; Anders Gummesson, MD, PhD; Olle Melander, MD, PhD; Sebastian Albinsson, PhD; Göran Dellgren, MD, PhD; Jan Borén, MD, PhD; Anders Jeppsson, MD, PhD; R. Thomas Lumbers, MD, PhD; Sonia Shah, PhD; Johan Nilsson, MD, PhD; Pradeep Natarajan, MD; Tuuli Lappalainen, PhD; Malin Levin, PhD; Hans Ehrencrona, MD, PhD; J. Gustav Smith, MD, PhD

# **Supplementary Information**

Supplementary Methods

Supplementary Results

Supplementary Tables

Supplementary Figures

# **Supplementary Methods**

## **Recruitment, genotyping and genotype quality control for the Malmö Diet and Cancer study**

The Malmö Diet and Cancer (MDC) study^1^ is a prospective population-based cohort recruited between 1991 and 1996 in Malmö, Sweden. Malmö is the third largest city in Sweden, and a neighbouring city to Lund, where most of the heart transplantations in this study were performed. At the time of recruitment, Malmö had a population of approximately 230,000 individuals. Letters of invitation were sent to all female citizens of Malmö born between 1923 and 1950, and all male citizens born between 1923 and 1945. A total of 30,447 individuals responded and underwent baseline examination, corresponding to a participation rate of approximately 40%. Anthropometric data, blood pressure, and a detailed questionnaire on medical history, as well as blood samples were collected. DNA was isolated from whole blood using a spin column-based method from Qiagen. Genome-wide genotyping of single nucleotide variants and exome sequencing was performed in the full cohort by Regeneron Genetics Centre.

Genotyping was performed using the Illumina Infinium Global Screening Array v1 (Illumina Inc., San Diego, CA, USA) according to the manufacturer’s standard protocols. Imputation was performed in three similarly sized batches to the Haplotype Reference Consortium reference panel for European populations (v1.1 from 2016) based on the Michigan imputation server (https://imputationserver.sph.umich.edu/index.html) with phasing based on the eagle software v2.3, resulting in a total number of 39,127,700 markers. Before imputation, autosomal SNPs with minor allele frequency >1%, with less than 1% missingness, with a Handy-Weinberg equilibrium p-value less than 1x10^-15^ passed the quality control. Individuals with more than 10% of missing genotype data were excluded, as were duplicated or sex discordant samples.

Exome capture was performed using an automated, high-throughput pipeline at Regeneron Genetics Center. Genomic DNA was enzymatically sheared into 200 base pair fragments and libraries created using a custom NEBNext Ultra II FS DNA library prep kit (New England Biolabs). Samples were pooled with xGen probes from Integrated DNA Technologies (IDT, Coralville, IA, USA). Captured fragments were bound to streptavidin-coupled Dynabeads (Thermo Fisher Scientific) and non-specific fragments removed through a series of washes according to instructions from the manufacturer (IDT). The captured DNA was amplified and quantified by quantitative polymerase chain reaction with KAPA HiFi polymerase (KAPA biosystems). The multiplexed samples were pooled and sequenced using 75 base paired-end reads on a Novaseq 6000 sequencer using S2 flow cells (Illumina Inc). A total of 38,997,831 bases were included in the targeted regions. More than 20x coverage was achieved over more than 90% of targeted bases in 99% of the samples.

The binary base call (bcl) files generated by the sequencers were transformed into fastq files by the bcl2fastq conversion software from Illumina. Reads were aligned to the human reference genome GRCh38 using the Burrows-Wheeler Aligner. Single nucleotide variants and insertion/deletions were called using the WeCall variant caller. Samples were excluded if disagreement between genetically determined and self-reported sex, homozygosity rates with D-stat >0.4, coverage of less than 20x achieved for less than 85% of targeted bases, genetically-inferred duplicate samples, or if sequencing variants were discordant with array calls.

The study was approved by the Regional Ethics Committee and all individuals provided written consent in accordance with the declaration of Helsinki.

## **DNA extraction, purification, whole genome sequencing, and data processing of the SwedeHeartSeq cohort**

Myocardial samples were sectioned and either snap-frozen (Gothenburg) or placed in RNAlater (Thermo Fisher Scientific, Waltham, MA, USA) and subsequently frozen (Lund) to -80°C, and remained frozen until thawing for DNA isolation. Genomic DNA was extracted and purified from explanted heart biopsies using a silica-based membrane method on spin columns in accordance with the manufacturers protocol (DNeasy Blood & Tissue kit, Qiagen, Venlo, the Netherlands). DNA quality was confirmed and quantified using a fluorometric method (Qubit, Thermo Fisher Scientific Inc., Waltham, MA, USA; OD260/280 of 1.8-2.0 was required for sequencing) and fragment length using an electrophoretic method (FragmentAnalyzer, Agilent Technologies Inc., Santa Clara, CA, USA). More than 3.5 ug of DNA were available from all patients. Sequencing was performed at the accredited national core facility SciLifeLab in Uppsala, Sweden. DNA libraries were constructed without polymerase chain reaction (PCR) amplification steps using the TruSeq PCR-free library prep kit (Illumina Inc.). Sequencing was performed to an average target sequencing depth of 60x using paired-end 150 bp sequencing in S4 flow cells with v1.5 reagent kits on a NovaSeq 6000 (Illumina Inc.) at the accredited national core facility SciLifeLab (National Genomics Infrastructure Uppsala/SNP&SEQ technology platform, SciLifeLab, Uppsala). Median coverage was 71x and coverage exceeded 30x at 90% of sites on average.

The binary base call (bcl) files generated by the sequencers were transformed into fastq files by the bcl2fastq conversion software from Illumina. Preprocessing, alignment, and variant calling was performed using the Sarek pipeline (v.2.7).^2^ Briefly, reads were aligned to the human reference genome GRCh38 using the Burrows-Wheeler Aligner. Duplicate read removal and quality score recalibration was performed using GATK (v.4.1.7.0).^3^ Variant calling for single nucleotide variants (SNVs) and insertions and deletions (indels) was performed using GATK HaplotypeCaller and Strelka (v.2.9.10).

## **Pathogenic variant annotation and prioritization pipeline**

Variant annotation and prioritization were performed using a slightly modified version of the Mutation Identification Pipeline (MIP) algorithm,^4^ which is an algorithm used for ranking variants according to predicted pathogenicity. This algorithm has been in use at the Clinical Genomics facility at SciLifeLab since 2014 and versions of it is in use at the major clinical genomics departments in Swedish healthcare and for research.^4-7^ The algorithm is based on output from the tools GATK, VEP, ANNOVAR and CADD and assigns each variant a pathogenicity score based on 8 categories of attributes as listed below. In the list below, categories as well as information on whether the maximum score (max), minimum score (min), or score summation (sum) are to be used. Scores from each category are then summed resulting in the final score. All variants with a total score of 15 or higher, with lower scores considered highly likely to represent benign variants based on clinical experience,^4,5^ were curated manually and classified according to ACMG criteria.^8^ Manual curation was also performed for all variants with a previous ClinVar submission classified as LP/P, regardless of pathogenicity score to ensure that no variant previously classified as pathogenic by ClinVar was missed.

1. **Variant call quality filter** (sum)
2. **Splicing** (max)
3. **Consequence** (max)
4. **Gene intolerance prediction** (max)
5. **Conservation** (sum)
6. **Protein prediction** (sum)
7. **Allele frequency** (min)
8. **Clinical significance** (sum)

#### 1. Variant Call Quality Score

All variants that pass the variant call quality filter in GATK HaplotypeCaller are assigned a score of 3.

| **Variant call quality** | **Score** |
| --- | --- |
| Not reported | 0 |
| . | 3 |
| PASS | 3 |

#### 2. Spliceogenicity Score

Spliceogenicity was assessed using MaxEntScan^9^ and SpliceAI.^10^ MaxEntScan scores were obtained via the MaxEntScan plugin in VEP and SpliceAI scores were obtained from CADD. The maximum variant score from these tools constitutes the Spliceogenicity Score.

| **MaxEntScan alternative score for native splice sites** | **Score** |
| --- | --- |
| Not reported | 0 |
| Low (>0, ≤6.2) | 3 |
| Medium (>6.2, ≤8.5) | 2 |
| High (>8.5) | 0 |

| **MaxEntScan difference score for native splice sites** | **Score** |
| --- | --- |
| Not reported | 0 |
| Low (≥0, ≤1.15) | 3 |
| High (>1.15) | 2 |

| **SpliceAI delta score acceptor gain** | **Score** |
| --- | --- |
| Not reported | 0 |
| Low (>0, <0.2) | 1 |
| Medium (≥0.2, <0.5) | 3 |
| High (≥0.5) | 5 |

| **SpliceAI delta score acceptor loss** | **Score** |
| --- | --- |
| Not reported | 0 |
| Low (>0, <0.2) | 1 |
| Medium (≥0.2, <0.5) | 3 |
| High (≥0.5) | 5 |

| **SpliceAI delta score donor gain** | **Score** |
| --- | --- |
| Not reported | 0 |
| Low (>0, <0.2) | 1 |
| Medium (≥0.2, <0.5) | 3 |
| High (≥0.5) | 5 |
| **SpliceAI delta score donor loss** | **Score** |
| Not reported | 0 |
| Low (>0, <0.2) | 1 |
| Medium (≥0.2, <0.5) | 3 |
| High (≥0.5) | 5 |

#### 3. Consequence Score

The consequence of the variant as predicted by VEP is assigned a Consequence Score. Some variants have multiple consequence terms, e.g., splice_donor_variant, and non_coding_transcript_variant, in which case the maximum consequence constitutes the Consequence Score.

| **Consequence** | **Score** |
| --- | --- |
| transcript_ablation | 10 |
| initiator_codon_variant | 9 |
| frameshift_variant | 8 |
| stop_gained | 8 |
| start_lost | 8 |
| stop_lost | 8 |
| splice_acceptor_variant | 8 |
| splice_donor_variant | 8 |
| inframe_deletion | 5 |
| transcript_amplification | 5 |
| splice_region_variant | 5 |
| missense_variant | 5 |
| protein_altering_variant | 5 |
| inframe_insertion | 5 |
| incomplete_terminal_codon_variant | 5 |
| non_coding_transcript_exon_variant | 3 |
| synonymous_variant | 2 |
| mature_mirna_variant | 1 |
| non_coding_transcript_variant | 1 |
| regulatory_region_variant | 1 |
| upstream_gene_variant | 1 |
| regulatory_region_amplification | 1 |
| tfbs_amplification | 1 |
| 5_prime_utr_variant | 1 |
| intron_variant | 1 |
| 3_prime_utr_variant | 1 |
| feature_truncation | 1 |
| TF_binding_site_variant | 1 |
| stop_retained_variant | 1 |
| feature_elongation | 1 |
| regulatory_region_ablation | 1 |
| tfbs_ablation | 1 |
| coding_sequence_variant | 1 |
| downstream_gene_variant | 1 |
| NMD_transcript_variant | 1 |
| intergenic_variant | 0 |
| Not reported | 0 |

#### 4. Gene Intolerance Score

The Gene Intolerance Score was obtained from LoFtool,^11^ which predicts gene intolerance based on the ratio of loss of function to synonymous variants in subjects from the ExAC database. The LoFtool score was obtained using a plugin to VEP.

| **LoFtool score** | **Score** |
| --- | --- |
| Not reported | 0 |
| High intolerance (≥0, ≤0.01) | 4 |
| Medium intolerance (>0.01, ≤0.1) | 2 |
| High intolerance (>0.1) | 0 |

#### 5. Conservation Score

Evolutionary locus conservation was assessed using three models, GERP++_RS score,^12^ phastCons score,^13^ and phyloP score.^14^ All three scores were obtained from ANNOVAR. The sum of all three scores constitutes the Conservation Score.

| **GERP++_RS score** | **Score** |
| --- | --- |
| Not reported | 0 |
| Not conserved (<2) | 0 |
| Conserved (≥2) | 1 |

| **phastCons100way_vertebrate score** | **Score** |
| --- | --- |
| Not reported | 0 |
| Not conserved (<0.8) | 0 |
| Conserved (≥0.8) | 1 |

| **phyloP100way_vertebrate** | **Score** |
| --- | --- |
| Not reported | 0 |
| Not conserved (<2.5) | 0 |
| Conserved (≥2.5) | 1 |

#### 6. Protein Prediction Score

Four prediction tools are used to assess variant deleteriousness, Polyphen,^15^ SIFT,^16^ REVEL,^17^ and CADD.^18^ The sum of the individual scores constitutes the Protein Prediction Score. Polyphen, SIFT and CADD phred scores were annotated using the CADD tool, REVEL was annotated using the REVEL plugin in VEP.

| **Polyphen value** | **Score** |
| --- | --- |
| Not reported | 0 |
| Benign (≤ 0.446) | 0 |
| Possibly damaging (> 0.446, < 0.908) | 1 |
| Probably damaging (≥ 0.908) | 1 |

| **SIFT** | **Score** |
| --- | --- |
| Not reported | 0 |
| Tolerated (≥ 0.05) | 0 |
| Deleterious (< 0.05) | 1 |

| **REVEL** | **Score** |
| --- | --- |
| Not reported | 0 |
| Tolerated (< 0.5) | 0 |
| Probably damaging (≥ 0.5, < 0.75) | 2 |
| Damaging (≥ 0.75) | 5 |

| **CADD** | **Score** |
| --- | --- |
| Not reported | 0 |
| Low (< 10) | 0 |
| Medium (≥ 10, < 20) | 2 |
| High (≥ 20, < 30) | 3 |
| Higher (≥ 30, < 40) | 4 |
| Highest (≥ 40) | 5 |

#### 7. Allele Frequency Score

We obtained allele frequencies from three reference cohorts using VEP (the 1000 genomes project, exome sequencing project of the National Heart Lung and Blood Institute (ESP), and gnomAD^19^). The maximum allele frequencies in African, admixed American, East Asian, European, and South Asian were used. We also obtained allele frequencies from exome sequencing of our local reference cohort (Malmö Diet and Cancer, MDCS). The minimum score of either the international cohorts (1000 genomes, ESP, gnomAD) or the MDCS was used as the Allele frequency score. Higher allele frequencies yielded lower scores as outlined below.

| **gnomAD allele frequency** | **Score** |
| --- | --- |
| Not reported | 4 |
| Very rare (> 0, ≤ 0.0005) | 3 |
| Rare (> 0.0005, ≤ 0.005) | 2 |
| Intermediate (> 0.005, ≤ 0.02) | 1 |
| Common (> 0.02) | -12 |

| **MDC allele frequency** | **Score** |
| --- | --- |
| Not reported | 4 |
| Very rare (> 0, ≤ 0.0005) | 3 |
| Rare (> 0.0005, ≤ 0.005) | 2 |
| Intermediate (> 0.005, ≤ 0.02) | 1 |
| Common (> 0.02) | -12 |

#### 8. ClinVar Score

All variants with a previous submission to ClinVar^20^ obtained a score based on a) clinical significance (the maximum value) and b) clinical review status (the maximum value). ClinVar variants were extracted on April 30^th^, 2022. The Clinical Review Status Score corresponds to the number of gold stars as assigned by ClinVar. The sum of the Clinical Significance Score and the Clinical Review Status Score constitutes the ClinVar Score.

| **ClinVar Clinical Significance** | **Score** |
| --- | --- |
| Not provided | 0 |
| Drug response | 0 |
| Other | 0 |
| Uncertain significance | 0 |
| Benign | -1 |
| Likely benign | 0 |
| Likely pathogenic | 5 |
| Pathogenic | 5 |

| **ClinVar Clinical Review Status** | **Score** |
| --- | --- |
| Not reported | 0 |
| No assertion provided | 0 |
| No assertion criteria provided | 0 |
| No interpretation for the single variant | 0 |
| Criteria provided, single submitter | 1 |
| Criteria provided, conflicting interpretations | 1 |
| Criteria provided, multiple submitters, no conflicts | 2 |
| Reviewed by expert panel | 3 |
| Practice guideline | 4 |

## **Gene collation**

We aimed to establish a gene list incorporating a broad set of genes that may be relevant to genetic forms of heart failure. Genes were collated from three main sources: curated genes by ClinGen, curated genes by PanelApp, and a systematic review of the literature. First, we compiled a list of genes curated by ClinGen as having moderate, strong, or definitive evidence (hereafter referred to as potentially pathogenic) for hypertrophic cardiomyopathy (HCM, including genes associated with clinical syndromes and genes causing HCM phenocopies),^21^ dilated cardiomyopathy (DCM),^22^ arrhythmogenic right ventricular cardiomyopathy (ARVC),^23^ and familial hypercholesterolemia (FH).^24^ Since we originally planned to analyse left ventricular non-compaction (LVNC) as a separate clinical entity, we also included articles with this subject as well. ClinGen has currently not published any gene curation for monogenic hypertension (MGH), LVNC, or restrictive cardiomyopathy (RCM). We supplemented this list with genes from PanelApp, an online crowd-sourcing tool created and moderated by Genomics England.^25^ Genes curated as amber or green (hereafter referred to as potentially pathogenic) were abstracted from the following panels:

1. Hypertrophic cardiomyopathy - teen and adult (v. 2.32), accessed 2021-11-18
2. Dilated cardiomyopathy - adult and teen (v. 1.26), accessed 2021-11-18
3. Arrhythmogenic cardiomyopathy (v 2.13), accessed 2021-11-18
4. Left Ventricular Noncompaction Cardiomyopathy (v. 1.4), accessed 2021-11-18
5. Cardiomyopathies - including childhood onset (v 1.59), accessed 2021-12-20
6. Familial hypercholesterolaemia - targeted panel (v. 1.9), accessed 2021-11-18

All genes that were classified as potentially pathogenic by either ClinGen or PanelApp were included. This list was supplemented with additional genes from a systematic literature review for genes associated with DCM, HCM, ARVC, LVNC, RCM, and MGH. Search phrases used are detailed below. All searches were performed with PubMed on November 1, 2021. Titles and abstracts yielded by the search were screened and articles that applied a systematic approach to review genes for each condition were included and genes included into the gene list. The following phrases were used:

1. Search for DCM: *"Cardiomyopathy, Dilated"[Mesh] AND (((gene) OR (genes)) OR (genet*)) AND (review[Filter]) AND (2015:2021/11/1[pdat]).* Search yielded 137 results of which 32 articles included a structured gene presentation and were included.
2. Search for HCM: *"Cardiomyopathy, Hypertrophic"[Mesh] AND (((gene) OR (genes)) OR (genet*)) AND (review[Filter]) AND (2015:2021/11/1[pdat]).* Search yielded 184 results of which 32 articles included a structured gene presentation and were included.
3. Search for ARVC: *"Arrhythmogenic Right Ventricular Dysplasia"[Mesh] AND (((gene) OR (genes)) OR (genet*)) AND (review[Filter]) AND (2015:2021/11/1[pdat]).* Search yielded 91 results of which 28 articles included a structured gene presentation and were included.
4. Search for RCM: *"Cardiomyopathy, Restrictive"[Mesh] AND (((gene) OR (genes)) OR (genet*)) AND (review[Filter]) AND (2015:2021/11/1[pdat]).* Search yielded 14 results of which 3 articles included a structured gene presentation and were included.
5. Search for LVNC: *"Isolated Noncompaction of the Ventricular Myocardium"[Mesh] AND (((gene) OR (genes)) OR (genet*)) AND (review[Filter]) AND (2015:2021/11/1[pdat]).* Search yielded 22 results of which 11 articles included a structured gene presentation and were included.
6. Search for MGH: *("Hypertension"[Mesh]) AND ((monogenic) OR (Mendelian) OR (autosomal) OR (recessive)) AND (review[Filter]) AND (2015:2021/11/1[pdat]).* Search yielded 105 results of which 16 articles included a structured gene presentation and were included.

In total, 105 unique articles were included from which 368 genes were extracted. In addition, one participant in our study had a prior diagnosis of glycogen storage disease type IV, carrying a variant in the gene *GBE1* which was added to the list also for a total of 369 genes (listed below in **Supplementary Fig.** and **Supplementary Table S1**).

## **Variant classification**

Manual classification was conducted in accordance with the American College of Medical Genetics (ACMG) criteria using gene- and condition-specific criteria when available.^8,26-28^ Variants were classified as benign/likely benign (B/LB), variant of uncertain significance (VUS), likely pathogenic (LP) and pathogenic (P). Variant interpretation was aided by the Alamut Visual Plus browser (Interactive Biosoftware, Rouen, France). All variants presented in this article were classified by board-certified clinical geneticists (MH and HE) in accordance with ACMG recommendations.^8^

## **Polygenic risk score derivation**

The HERMES dataset used for development of the risk score included 153,174 HF cases and 1,793,175 controls from a meta-analysis of longitudinal population-based cohorts, hospital-based electronic health record cohorts, case-control studies, and clinical trials. Protocols for quality control and meta-analysis in this consortium have been described previously.^29^

Polygenic risk scores were derived based on summary statistics from the HERMES dataset using the LDpred2 algorithm as implemented in the R package bigsnpr 1.8.1 in R version 3.6.0 (R Foundation, Vienna, Austria).^30^ The ‘auto’ option in LDpred2 was used, which directly learns the two LDpred2 parameters (sparsity and SNP heritability) from the dataset. The correlation of polymorphisms (linkage disequilibrium) was obtained from a reference panel provided by the software which is based on the UK Biobank and 1000 Genomes Project samples. Analyses were restricted to variants with minor allele frequency>0.01, and Hardy-Weinberg equilibrium *P*<10^-10^.

# **Supplementary results**

## **Overall yield of genetic testing for monogenic variants**

A total of 22,393,708 single nucleotide variants and indels were identified from WGS in the 101 early-onset HF cases, with a median of 5,028,520 variants per individual (interquartile range 5,007,377-5,057,396). Of these, 243,634 variants were located in the 369 genes from our early-onset HF list with a median of 50,673 per individual (49,816-51,353). Our pathogenicity annotation pipeline identified 1,059 of these variants with a pathogenicity score of 15 or higher that were prioritized for manual classification. Of these, manual classification according to ACMG criteria resulted in 71 (6.7%) being classified as LP/P, out of which 39 (3.7%) were categorized as contributing to the observed phenotype whereas the other 32 (3.0%) were heterozygous variants in autosomal recessive genes or pathogenic variants not consistent with exhibited phenotype. Conversely, 450 variants (42.4%) were classified as LB/B. The remaining 538 variants were classified as VUS (50.8%), of which 13 (1.2%) were identified as having suggestive evidence of contributing to phenotype during manual curation. From the VUS group, 103 were missense variants in the *TTN* gene.

Of the 39 LP/P variants, at least one was identified in each of 34 individuals (34%), while at least one VUS was present in almost all (100, 99%) and at least one VUS with suggestive evidence of pathogenicity in 11 individuals (11%). All LP/P and VUS variants with suggestive evidence are listed in **Supplementary Table S4** (manually curated variants classified as not significant are not presented). Four of the 39 LP/P variants (two in *POLG*, one in *TTR*, and one in *KCNQ1*) and none of the 11 VUS variants with suggestive evidence of pathogenicity (*DSG2*) were present in the local population-based cohort MDCS, although at sufficiently low frequency to not reduce pathogenicity scores and with a lower prevalence than the maximum ancestry-based allele frequency in gnomAD.

Four individuals had more than one LP/P variant concurrently, however, in three of these cases, one or two of the variants was interpreted as non-Mendelian, low-penetrance risk factors (in *MYL3*, *MYBPC3* and *POLG* in carriers of pathogenic *TNNT2*, *MYBPC3* and *TTR* variants respectively) while in one case two *GYG1* variants were interpreted as compound heterozygous.

Two individuals had one pathogenic variant and one VUS of suggestive evidence: the patient with a previous diagnosis of glycogen storage disease IV and a pathogenic variant in *GBE1* carried a VUS in the same gene, and one patient with ARVC and a pathogenic variant in *DSG2* carried a VUS in the same gene.

# **References**

1. Smith JG, Platonov PG, Hedblad B, Engstrom G, Melander O. Atrial fibrillation in the Malmo Diet and Cancer study: a study of occurrence, risk factors and diagnostic validity. *Eur J Epidemiol* 2010;**25**:95-102. doi: 10.1007/s10654-009-9404-1

2. Garcia M, Juhos S, Larsson M*, et al.* Sarek: A portable workflow for whole-genome sequencing analysis of germline and somatic variants. *F1000Res* 2020;**9**:63. doi: 10.12688/f1000research.16665.2

3. McKenna A, Hanna M, Banks E*, et al.* The Genome Analysis Toolkit: a MapReduce framework for analyzing next-generation DNA sequencing data. *Genome Res* 2010;**20**:1297-1303. doi: 10.1101/gr.107524.110

4. Stranneheim H, Engvall M, Naess K*, et al.* Rapid pulsed whole genome sequencing for comprehensive acute diagnostics of inborn errors of metabolism. *BMC Genomics* 2014;**15**:1090. doi: 10.1186/1471-2164-15-1090

5. Stranneheim H, Lagerstedt-Robinson K, Magnusson M*, et al.* Integration of whole genome sequencing into a healthcare setting: high diagnostic rates across multiple clinical entities in 3219 rare disease patients. *Genome Med* 2021;**13**:40. doi: 10.1186/s13073-021-00855-5

6. Haack TB, Ignatius E, Calvo-Garrido J*, et al.* Absence of the Autophagy Adaptor SQSTM1/p62 Causes Childhood-Onset Neurodegeneration with Ataxia, Dystonia, and Gaze Palsy. *Am J Hum Genet* 2016;**99**:735-743. doi: 10.1016/j.ajhg.2016.06.026

7. Zhao S, Zhang Y, Hallgrimsdottir S*, et al.* Expanding the mutation and phenotype spectrum of MYH3-associated skeletal disorders. *NPJ Genom Med* 2022;**7**:11. doi: 10.1038/s41525-021-00273-x

8. Richards S, Aziz N, Bale S*, et al.* Standards and guidelines for the interpretation of sequence variants: a joint consensus recommendation of the American College of Medical Genetics and Genomics and the Association for Molecular Pathology. *Genet Med* 2015;**17**:405-424. doi: 10.1038/gim.2015.30

9. Shamsani J, Kazakoff SH, Armean IM*, et al.* A plugin for the Ensembl Variant Effect Predictor that uses MaxEntScan to predict variant spliceogenicity. *Bioinformatics* 2019;**35**:2315-2317. doi: 10.1093/bioinformatics/bty960

10. Jaganathan K, Kyriazopoulou Panagiotopoulou S, McRae JF*, et al.* Predicting Splicing from Primary Sequence with Deep Learning. *Cell* 2019;**176**:535-548 e524. doi: 10.1016/j.cell.2018.12.015

11. Fadista J, Oskolkov N, Hansson O, Groop L. LoFtool: a gene intolerance score based on loss-of-function variants in 60 706 individuals. *Bioinformatics* 2017;**33**:471-474. doi: 10.1093/bioinformatics/btv602

12. Davydov EV, Goode DL, Sirota M*, et al.* Identifying a high fraction of the human genome to be under selective constraint using GERP++. *PLoS Comput Biol* 2010;**6**:e1001025. doi: 10.1371/journal.pcbi.1001025

13. Siepel A, Bejerano G, Pedersen JS*, et al.* Evolutionarily conserved elements in vertebrate, insect, worm, and yeast genomes. *Genome Res* 2005;**15**:1034-1050. doi: 10.1101/gr.3715005

14. Pollard KS, Hubisz MJ, Rosenbloom KR, Siepel A. Detection of nonneutral substitution rates on mammalian phylogenies. *Genome Res* 2010;**20**:110-121. doi: 10.1101/gr.097857.109

15. Ramensky V, Bork P, Sunyaev S. Human non-synonymous SNPs: server and survey. *Nucleic Acids Res* 2002;**30**:3894-3900. doi: 10.1093/nar/gkf493

16. Ng PC, Henikoff S. SIFT: Predicting amino acid changes that affect protein function. *Nucleic Acids Res* 2003;**31**:3812-3814. doi: 10.1093/nar/gkg509

17. Ioannidis NM, Rothstein JH, Pejaver V*, et al.* REVEL: An Ensemble Method for Predicting the Pathogenicity of Rare Missense Variants. *Am J Hum Genet* 2016;**99**:877-885. doi: 10.1016/j.ajhg.2016.08.016

18. Rentzsch P, Witten D, Cooper GM, Shendure J, Kircher M. CADD: predicting the deleteriousness of variants throughout the human genome. *Nucleic Acids Res* 2019;**47**:D886-D894. doi: 10.1093/nar/gky1016

19. Karczewski KJ, Francioli LC, Tiao G*, et al.* The mutational constraint spectrum quantified from variation in 141,456 humans. *Nature* 2020;**581**:434-443. doi: 10.1038/s41586-020-2308-7

20. Landrum MJ, Lee JM, Benson M*, et al.* ClinVar: improving access to variant interpretations and supporting evidence. *Nucleic Acids Res* 2018;**46**:D1062-D1067. doi: 10.1093/nar/gkx1153

21. Ingles J, Goldstein J, Thaxton C*, et al.* Evaluating the Clinical Validity of Hypertrophic Cardiomyopathy Genes. *Circ Genom Precis Med* 2019;**12**:e002460. doi: 10.1161/CIRCGEN.119.002460

22. Jordan E, Peterson L, Ai T*, et al.* Evidence-Based Assessment of Genes in Dilated Cardiomyopathy. *Circulation* 2021;**144**:7-19. doi: 10.1161/CIRCULATIONAHA.120.053033

23. James CA, Jongbloed JDH, Hershberger RE*, et al.* International Evidence Based Reappraisal of Genes Associated With Arrhythmogenic Right Ventricular Cardiomyopathy Using the Clinical Genome Resource Framework. *Circ Genom Precis Med* 2021;**14**:e003273. doi: 10.1161/CIRCGEN.120.003273

24. Iacocca MA, Chora JR, Carrie A*, et al.* ClinVar database of global familial hypercholesterolemia-associated DNA variants. *Hum Mutat* 2018;**39**:1631-1640. doi: 10.1002/humu.23634

25. Martin AR, Williams E, Foulger RE*, et al.* PanelApp crowdsources expert knowledge to establish consensus diagnostic gene panels. *Nat Genet* 2019;**51**:1560-1565. doi: 10.1038/s41588-019-0528-2

26. Morales A, Kinnamon DD, Jordan E*, et al.* Variant Interpretation for Dilated Cardiomyopathy: Refinement of the American College of Medical Genetics and Genomics/ClinGen Guidelines for the DCM Precision Medicine Study. *Circ Genom Precis Med* 2020;**13**:e002480. doi: 10.1161/CIRCGEN.119.002480

27. Chora JR, Iacocca MA, Tichy L*, et al.* The Clinical Genome Resource (ClinGen) Familial Hypercholesterolemia Variant Curation Expert Panel consensus guidelines for LDLR variant classification. *Genet Med* 2022;**24**:293-306. doi: 10.1016/j.gim.2021.09.012

28. Kelly MA, Caleshu C, Morales A*, et al.* Adaptation and validation of the ACMG/AMP variant classification framework for MYH7-associated inherited cardiomyopathies: recommendations by ClinGen's Inherited Cardiomyopathy Expert Panel. *Genet Med* 2018;**20**:351-359. doi: 10.1038/gim.2017.218

29. Lumbers RT, Shah S, Lin H*, et al.* The genomics of heart failure: design and rationale of the HERMES consortium. *ESC Heart Fail* 2021;**8**:5531-5541. doi: 10.1002/ehf2.13517

30. Prive F, Arbel J, Vilhjalmsson BJ. LDpred2: better, faster, stronger. *Bioinformatics* 2020;**36**:5424-5431. doi: 10.1093/bioinformatics/btaa1029

31. Roberts AM, Ware JS, Herman DS*, et al.* Integrated allelic, transcriptional, and phenomic dissection of the cardiac effects of titin truncations in health and disease. *Sci Transl Med* 2015;**7**:270ra276. doi: 10.1126/scitranslmed.3010134

# **Supplementary Tables**

**Table S1.** References for all genes in **Fig. S1** associated with monogenic heart muscle disease in systematic literature review with references. Aside from the literature review, six references are online references from PanelApp, and references 3, 21, 93, and 96 are position papers from ClinGen.

| 1. Peters S, Johnson R, Birch S, Zentner D, Hershberger RE, Fatkin D. Familial Dilated Cardiomyopathy. *Heart Lung Circ*. 2020;29:566-574. doi: 10.1016/j.hlc.2019.11.018  2. Genomics England PanelApp. Cardiomyopathies - including childhood onset (v. 1.59) [Internet]. 2021 [accessed November 18, 2021]. Available from: https://panelapp.genomicsengland.co.uk/panels/749/.  3. Ingles J, Goldstein J, Thaxton C, Caleshu C, Corty EW, Crowley SB, Dougherty K, Harrison SM, McGlaughon J, Milko LV, et al. Evaluating the Clinical Validity of Hypertrophic Cardiomyopathy Genes. *Circ Genom Precis Med*. 2019;12:e002460. doi: 10.1161/CIRCGEN.119.002460  4. Stafford F, Thomson K, Butters A, Ingles J. Hypertrophic Cardiomyopathy: Genetic Testing and Risk Stratification. *Curr Cardiol Rep*. 2021;23:9. doi: 10.1007/s11886-020-01437-4  5. Baulina NM, Kiselev IS, Chumakova OS, Favorova OO. [Hypertrophic Cardiomyopathy as an Oligogenic Disease: Transcriptomic Arguments]. *Mol Biol (Mosk)*. 2020;54:955-967. doi: 10.31857/S0026898420060026  6. Ahluwalia M, Ho CY. Cardiovascular genetics: the role of genetic testing in diagnosis and management of patients with hypertrophic cardiomyopathy. *Heart*. 2021;107:183-189. doi: 10.1136/heartjnl-2020-316798  7. Batzner A, Seggewiss H. [Hypertrophic cardiomyopathy]. *Herz*. 2020;45:233-242. doi: 10.1007/s00059-020-04899-y  8. Popa-Fotea NM, Micheu MM, Bataila V, Scafa-Udriste A, Dorobantu L, Scarlatescu AI, Zamfir D, Stoian M, Onciul S, Dorobantu M. Exploring the Continuum of Hypertrophic Cardiomyopathy-From DNA to Clinical Expression. *Medicina (Kaunas)*. 2019;55. doi: 10.3390/medicina55060299  9. Geske JB, Ommen SR, Gersh BJ. Hypertrophic Cardiomyopathy: Clinical Update. *JACC Heart Fail*. 2018;6:364-375. doi: 10.1016/j.jchf.2018.02.010  10. Cao Y, Zhang PY. Review of recent advances in the management of hypertrophic cardiomyopathy. *Eur Rev Med Pharmacol Sci*. 2017;21:5207-5210. doi: 10.26355/eurrev_201711_13841  11. Ren X, Hensley N, Brady MB, Gao WD. The Genetic and Molecular Bases for Hypertrophic Cardiomyopathy: The Role for Calcium Sensitization. *J Cardiothorac Vasc Anesth*. 2018;32:478-487. doi: 10.1053/j.jvca.2017.05.035  12. Ueda Y, Stern JA. A One Health Approach to Hypertrophic Cardiomyopathy. *Yale J Biol Med*. 2017;90:433-448.  13. Sartorio CL, Lazzeroni D, Bertoli G, Camici PG. Theranostic biomarkers in hypertrophic cardiomyopathy: insights in a long road ahead. *Front Biosci (Landmark Ed)*. 2017;22:1724-1749. doi: 10.2741/4568  14. Czepluch F, Hasenfuss G, Wollnik B. [Modern genetic counselling : Practical aspects exemplified by hypertrophic cardiomyopathy]. *Internist (Berl)*. 2018;59:790-798. doi: 10.1007/s00108-018-0452-z  15. Alejandra Restrepo-Cordoba M, Campuzano O, Ripoll-Vera T, Cobo-Marcos M, Mademont-Soler I, Gamez JM, Dominguez F, Gonzalez-Lopez E, Padron-Barthe L, Lara-Pezzi E, et al. Usefulness of Genetic Testing in Hypertrophic Cardiomyopathy: an Analysis Using Real-World Data. *J Cardiovasc Transl Res*. 2017;10:35-46. doi: 10.1007/s12265-017-9730-8  16. Viola HM, Hool LC. Impaired calcium handling and mitochondrial metabolic dysfunction as early markers of hypertrophic cardiomyopathy. *Arch Biochem Biophys*. 2019;665:166-174. doi: 10.1016/j.abb.2019.03.006  17. Veselka J, Anavekar NS, Charron P. Hypertrophic obstructive cardiomyopathy. *Lancet*. 2017;389:1253-1267. doi: 10.1016/S0140-6736(16)31321-6  18. Raghow R. An 'Omics' Perspective on Cardiomyopathies and Heart Failure. *Trends Mol Med*. 2016;22:813-827. doi: 10.1016/j.molmed.2016.07.007  19. Kuusisto J, Sipola P, Jaaskelainen P, Naukkarinen A. Current perspectives in hypertrophic cardiomyopathy with the focus on patients in the Finnish population: a review. *Ann Med*. 2016;48:496-508. doi: 10.1080/07853890.2016.1187764  20. Gomez J, Reguero JR, Coto E. The Ups and Downs of Genetic Diagnosis of Hypertrophic Cardiomyopathy. *Rev Esp Cardiol (Engl Ed)*. 2016;69:61-68. doi: 10.1016/j.rec.2015.10.001  21. Jordan E, Peterson L, Ai T, Asatryan B, Bronicki L, Brown E, Celeghin R, Edwards M, Fan J, Ingles J, et al. Evidence-Based Assessment of Genes in Dilated Cardiomyopathy. *Circulation*. 2021;144:7-19. doi: 10.1161/CIRCULATIONAHA.120.053033  22. Tayal U, Ware JS, Lakdawala NK, Heymans S, Prasad SK. Understanding the genetics of adult-onset dilated cardiomyopathy: what a clinician needs to know. *Eur Heart J*. 2021;42:2384-2396. doi: 10.1093/eurheartj/ehab286  23. Genomics England PanelApp. Dilated cardiomyopathy - teen and adult (v. 1.26) [Internet]. 2021 [accessed November 18, 2021]. Available from: https://panelapp.genomicsengland.co.uk/panels/652/.  24. Jordan E, Hershberger RE. Considering complexity in the genetic evaluation of dilated cardiomyopathy. *Heart*. 2021;107:106-112. doi: 10.1136/heartjnl-2020-316658  25. Wilsbacher LD. Clinical Implications of the Genetic Architecture of Dilated Cardiomyopathy. *Curr Cardiol Rep*. 2020;22:170. doi: 10.1007/s11886-020-01423-w  26. Hanselmann A, Veltmann C, Bauersachs J, Berliner D. Dilated cardiomyopathies and non-compaction cardiomyopathy. *Herz*. 2020;45:212-220. doi: 10.1007/s00059-020-04903-5  27. Lamounier Junior A, Ferrari F, Max R, Ritt LEF, Stein R. Importance of Genetic Testing in Dilated Cardiomyopathy: Applications and Challenges in Clinical Practice. *Arq Bras Cardiol*. 2019;113:274-281. doi: 10.5935/abc.20190144  28. Bakalakos A, Ritsatos K, Anastasakis A. Current perspectives on the diagnosis and management of dilated cardiomyopathy Beyond heart failure: a Cardiomyopathy Clinic Doctor's point of view. *Hellenic J Cardiol*. 2018;59:254-261. doi: 10.1016/j.hjc.2018.05.008  29. Bondue A, Arbustini E, Bianco A, Ciccarelli M, Dawson D, De Rosa M, Hamdani N, Hilfiker-Kleiner D, Meder B, Leite-Moreira AF, et al. Complex roads from genotype to phenotype in dilated cardiomyopathy: scientific update from the Working Group of Myocardial Function of the European Society of Cardiology. *Cardiovasc Res*. 2018;114:1287-1303. doi: 10.1093/cvr/cvy122  30. McNally EM, Mestroni L. Dilated Cardiomyopathy: Genetic Determinants and Mechanisms. *Circ Res*. 2017;121:731-748. doi: 10.1161/CIRCRESAHA.116.309396  31. de Gonzalo-Calvo D, Quezada M, Campuzano O, Perez-Serra A, Broncano J, Ayala R, Ramos M, Llorente-Cortes V, Blasco-Turrion S, Morales FJ, et al. Familial dilated cardiomyopathy: A multidisciplinary entity, from basic screening to novel circulating biomarkers. *Int J Cardiol*. 2017;228:870-880. doi: 10.1016/j.ijcard.2016.11.045  32. Favalli V, Serio A, Grasso M, Arbustini E. Genetic causes of dilated cardiomyopathy. *Heart*. 2016;102:2004-2014. doi: 10.1136/heartjnl-2015-308190  33. Japp AG, Gulati A, Cook SA, Cowie MR, Prasad SK. The Diagnosis and Evaluation of Dilated Cardiomyopathy. *J Am Coll Cardiol*. 2016;67:2996-3010. doi: 10.1016/j.jacc.2016.03.590  34. Muchtar E, Blauwet LA, Gertz MA. Restrictive Cardiomyopathy: Genetics, Pathogenesis, Clinical Manifestations, Diagnosis, and Therapy. *Circ Res*. 2017;121:819-837. doi: 10.1161/CIRCRESAHA.117.310982  35. Kubik M, Dabrowska-Kugacka A, Lewicka E, Danilowicz-Szymanowicz L, Raczak G. Predictors of poor outcome in patients with left ventricular noncompaction: Review of the literature. *Adv Clin Exp Med*. 2018;27:415-422. doi: 10.17219/acem/67457  36. Dong X, Fan P, Tian T, Yang Y, Xiao Y, Yang K, Liu Y, Zhou X. Recent advancements in the molecular genetics of left ventricular noncompaction cardiomyopathy. *Clin Chim Acta*. 2017;465:40-44. doi: 10.1016/j.cca.2016.12.013  37. Arbustini E, Favalli V, Narula N, Serio A, Grasso M. Left Ventricular Noncompaction: A Distinct Genetic Cardiomyopathy? *J Am Coll Cardiol*. 2016;68:949-966. doi: 10.1016/j.jacc.2016.05.096  38. Captur G, Syrris P, Obianyo C, Limongelli G, Moon JC. Formation and Malformation of Cardiac Trabeculae: Biological Basis, Clinical Significance, and Special Yield of Magnetic Resonance Imaging in Assessment. *Can J Cardiol*. 2015;31:1325-1337. doi: 10.1016/j.cjca.2015.07.003  39. Hussein A, Karimianpour A, Collier P, Krasuski RA. Isolated Noncompaction of the Left Ventricle in Adults. *J Am Coll Cardiol*. 2015;66:578-585. doi: 10.1016/j.jacc.2015.06.017  40. Ting TW, Jamuar SS, Brett MS, Tan ES, Cham BW, Lim JY, Law HY, Tan EC, Choo JT, Lai AH. Left Ventricular Non-compaction: Is It Genetic? *Pediatr Cardiol*. 2015;36:1565-1572. doi: 10.1007/s00246-015-1222-5  41. Hayashi T. Hypertrophic Cardiomyopathy: Diverse Pathophysiology Revealed by Genetic Research, Toward Future Therapy. *Keio J Med*. 2020;69:77-87. doi: 10.2302/kjm.2019-0012-OA  42. Perez-Serra A, Toro R, Sarquella-Brugada G, de Gonzalo-Calvo D, Cesar S, Carro E, Llorente-Cortes V, Iglesias A, Brugada J, Brugada R, et al. Genetic basis of dilated cardiomyopathy. *Int J Cardiol*. 2016;224:461-472. doi: 10.1016/j.ijcard.2016.09.068  43. Mazzarotto F, Olivotto I, Boschi B, Girolami F, Poggesi C, Barton PJR, Walsh R. Contemporary Insights Into the Genetics of Hypertrophic Cardiomyopathy: Toward a New Era in Clinical Testing? *J Am Heart Assoc*. 2020;9:e015473. doi: 10.1161/JAHA.119.015473  44. Streltsova AA, Gudkova AY, Kostareva AA. [Left ventricular non - compaction: contemporary view of genetic background, clinical course, diagnostic and treatment]. *Ter Arkh*. 2019;91:90-97. doi: 10.26442/00403660.2019.12.000142  45. Araco M, Merlo M, Carr-White G, Sinagra G. Genetic bases of dilated cardiomyopathy. *J Cardiovasc Med (Hagerstown)*. 2017;18:123-130. doi: 10.2459/JCM.0000000000000432  46. Cannie D, Elliott P. The genetics of left ventricular noncompaction. *Curr Opin Cardiol*. 2021;36:301-308. doi: 10.1097/HCO.0000000000000844  47. Chen SN, Mestroni L, Taylor MRG. Genetics of dilated cardiomyopathy. *Curr Opin Cardiol*. 2021;36:288-294. doi: 10.1097/HCO.0000000000000845  48. Cho KW, Lee J, Kim Y. Genetic Variations Leading to Familial Dilated Cardiomyopathy. *Mol Cells*. 2016;39:722-727. doi: 10.14348/molcells.2016.0061  49. Bonaventura J, Polakova E, Vejtasova V, Veselka J. Genetic Testing in Patients with Hypertrophic Cardiomyopathy. *Int J Mol Sci*. 2021;22. doi: 10.3390/ijms221910401  50. Filho DCS, do Rego Aquino PL, de Souza Silva G, Fabro CB. Left Ventricular Noncompaction: New Insights into a Poorly Understood Disease. *Curr Cardiol Rev*. 2021;17:209-216. doi: 10.2174/1573403X16666200716151015  51. Rooms I, Dujardin K, De Sutter J. Non-compaction cardiomyopathy: a genetically and clinically heterogeneous disorder. *Acta Cardiol*. 2015;70:625-631. doi: 10.2143/AC.70.6.3120173  52. Genomics England PanelApp. Hypertrophic cardiomyopathy - teen and adult (v. 2.32) [Internet]. 2021 [accessed November 18, 2021]. Available from: https://panelapp.genomicsengland.co.uk/panels/49/.  53. Genomics England PanelApp. Left Ventricular Noncompaction Cardiomyopathy (v. 1.4) [Internet]. 2021 [accessed November 18, 2021]. Available from: https://panelapp.genomicsengland.co.uk/panels/238/.  54. Hu CS. A comprehensive strategy for managing arrhythmogenic right ventricular cardiomyopathy. *Turk Kardiyol Dern Ars*. 2020;48:88-95. doi: 10.5543/tkda.2019.74184  55. Merlo M, Cappelletto C, De Angelis G, Porcari A, Caiffa T, Lardieri G, Pagnan L, Severini GM, Dal Ferro M, Stolfo D, et al. [Diagnostic work-up and clinical management of cardiomyopathies: the operative protocol from the Cardiothoracovascular Department of Trieste, Italy]. *G Ital Cardiol (Rome)*. 2020;21:935-953. doi: 10.1714/3472.34548  56. Tayal U, Prasad S, Cook SA. Genetics and genomics of dilated cardiomyopathy and systolic heart failure. *Genome Med*. 2017;9:20. doi: 10.1186/s13073-017-0410-8  57. Sabater-Molina M, Perez-Sanchez I, Hernandez Del Rincon JP, Gimeno JR. Genetics of hypertrophic cardiomyopathy: A review of current state. *Clin Genet*. 2018;93:3-14. doi: 10.1111/cge.13027  58. Young L, Smedira NG, Tower-Rader A, Lever H, Desai MY. Hypertrophic cardiomyopathy: A complex disease. *Cleve Clin J Med*. 2018;85:399-411. doi: 10.3949/ccjm.85a.17076  59. Reichart D, Magnussen C, Zeller T, Blankenberg S. Dilated cardiomyopathy: from epidemiologic to genetic phenotypes: A translational review of current literature. *J Intern Med*. 2019;286:362-372. doi: 10.1111/joim.12944  60. Weintraub RG, Semsarian C, Macdonald P. Dilated cardiomyopathy. *Lancet*. 2017;390:400-414. doi: 10.1016/S0140-6736(16)31713-5  61. Cimiotti D, Budde H, Hassoun R, Jaquet K. Genetic Restrictive Cardiomyopathy: Causes and Consequences-An Integrative Approach. *Int J Mol Sci*. 2021;22. doi: 10.3390/ijms22020558  62. Marrow BA, Cook SA, Prasad SK, McCann GP. Emerging Techniques for Risk Stratification in Nonischemic Dilated Cardiomyopathy: JACC Review Topic of the Week. *J Am Coll Cardiol*. 2020;75:1196-1207. doi: 10.1016/j.jacc.2019.12.058  63. Rosenbaum AN, Agre KE, Pereira NL. Genetics of dilated cardiomyopathy: practical implications for heart failure management. *Nat Rev Cardiol*. 2020;17:286-297. doi: 10.1038/s41569-019-0284-0  64. Schultheiss HP, Fairweather D, Caforio ALP, Escher F, Hershberger RE, Lipshultz SE, Liu PP, Matsumori A, Mazzanti A, McMurray J, et al. Dilated cardiomyopathy. *Nat Rev Dis Primers*. 2019;5:32. doi: 10.1038/s41572-019-0084-1  65. Yadav S, Sitbon YH, Kazmierczak K, Szczesna-Cordary D. Hereditary heart disease: pathophysiology, clinical presentation, and animal models of HCM, RCM, and DCM associated with mutations in cardiac myosin light chains. *Pflugers Arch*. 2019;471:683-699. doi: 10.1007/s00424-019-02257-4  66. Peters S, Kumar S, Elliott P, Kalman JM, Fatkin D. Arrhythmic Genotypes in Familial Dilated Cardiomyopathy: Implications for Genetic Testing and Clinical Management. *Heart Lung Circ*. 2019;28:31-38. doi: 10.1016/j.hlc.2018.09.010  67. Fu Y, Eisen HJ. Genetics of Dilated Cardiomyopathy. *Curr Cardiol Rep*. 2018;20:121. doi: 10.1007/s11886-018-1061-0  68. Paldino A, De Angelis G, Merlo M, Gigli M, Dal Ferro M, Severini GM, Mestroni L, Sinagra G. Genetics of Dilated Cardiomyopathy: Clinical Implications. *Curr Cardiol Rep*. 2018;20:83. doi: 10.1007/s11886-018-1030-7  69. Patel V, Asatryan B, Siripanthong B, Munroe PB, Tiku-Owens A, Lopes LR, Khanji MY, Protonotarios A, Santangeli P, Muser D, et al. State of the Art Review on Genetics and Precision Medicine in Arrhythmogenic Cardiomyopathy. *Int J Mol Sci*. 2020;21. doi: 10.3390/ijms21186615  70. James CA, Syrris P, van Tintelen JP, Calkins H. The role of genetics in cardiovascular disease: arrhythmogenic cardiomyopathy. *Eur Heart J*. 2020;41:1393-1400. doi: 10.1093/eurheartj/ehaa141  71. Paul M, Schulze-Bahr E. Arrhythmogenic right ventricular cardiomyopathy : Evolving from unique clinical features to a complex pathophysiological concept. *Herz*. 2020;45:243-251. doi: 10.1007/s00059-020-04907-1  72. Elliott PM, Anastasakis A, Asimaki A, Basso C, Bauce B, Brooke MA, Calkins H, Corrado D, Duru F, Green KJ, et al. Definition and treatment of arrhythmogenic cardiomyopathy: an updated expert panel report. *Eur J Heart Fail*. 2019;21:955-964. doi: 10.1002/ejhf.1534  73. Gandjbakhch E, Redheuil A, Pousset F, Charron P, Frank R. Clinical Diagnosis, Imaging, and Genetics of Arrhythmogenic Right Ventricular Cardiomyopathy/Dysplasia: JACC State-of-the-Art Review. *J Am Coll Cardiol*. 2018;72:784-804. doi: 10.1016/j.jacc.2018.05.065  74. Wang W, James CA, Calkins H. Diagnostic and therapeutic strategies for arrhythmogenic right ventricular dysplasia/cardiomyopathy patient. *Europace*. 2019;21:9-21. doi: 10.1093/europace/euy063  75. Hoorntje ET, Te Rijdt WP, James CA, Pilichou K, Basso C, Judge DP, Bezzina CR, van Tintelen JP. Arrhythmogenic cardiomyopathy: pathology, genetics, and concepts in pathogenesis. *Cardiovasc Res*. 2017;113:1521-1531. doi: 10.1093/cvr/cvx150  76. Corrado D, Basso C, Judge DP. Arrhythmogenic Cardiomyopathy. *Circ Res*. 2017;121:784-802. doi: 10.1161/CIRCRESAHA.117.309345  77. Mazurek S, Kim GH. Genetic and epigenetic regulation of arrhythmogenic cardiomyopathy. *Biochim Biophys Acta Mol Basis Dis*. 2017;1863:2064-2069. doi: 10.1016/j.bbadis.2017.04.020  78. Haugaa KH, Haland TF, Leren IS, Saberniak J, Edvardsen T. Arrhythmogenic right ventricular cardiomyopathy, clinical manifestations, and diagnosis. *Europace*. 2016;18:965-972. doi: 10.1093/europace/euv340  79. McGregor SM, Husain AN. A Brief Review and Update of the Clinicopathologic Diagnosis of Arrhythmogenic Cardiomyopathy. *Arch Pathol Lab Med*. 2015;139:1181-1186. doi: 10.5858/arpa.2014-0114-RS  80. Que D, Yang P, Song X, Liu L. Traditional vs. genetic pathogenesis of arrhythmogenic right ventricular cardiomyopathy. *Europace*. 2015;17:1770-1776. doi: 10.1093/europace/euv042  81. Alcalde M, Campuzano O, Sarquella-Brugada G, Arbelo E, Allegue C, Partemi S, Iglesias A, Oliva A, Brugada J, Brugada R. Clinical interpretation of genetic variants in arrhythmogenic right ventricular cardiomyopathy. *Clin Res Cardiol*. 2015;104:288-303. doi: 10.1007/s00392-014-0794-z  82. Genomics England PanelApp. Arrhythmogenic cardiomyopathy (v. 2.13) [Internet]. 2021 [accessed November 18, 2021]. Available from: https://panelapp.genomicsengland.co.uk/panels/134/.  83. Stadiotti I, Pompilio G, Maione AS, Pilato CA, D'Alessandra Y, Sommariva E. Arrhythmogenic cardiomyopathy: what blood can reveal? *Heart Rhythm*. 2019;16:470-477. doi: 10.1016/j.hrthm.2018.09.023  84. Moncayo-Arlandi J, Brugada R. Unmasking the molecular link between arrhythmogenic cardiomyopathy and Brugada syndrome. *Nat Rev Cardiol*. 2017;14:744-756. doi: 10.1038/nrcardio.2017.103  85. Basso C, Pilichou K, Bauce B, Corrado D, Thiene G. Diagnostic Criteria, Genetics, and Molecular Basis of Arrhythmogenic Cardiomyopathy. *Heart Fail Clin*. 2018;14:201-213. doi: 10.1016/j.hfc.2018.01.002  86. Cipriani A, Perazzolo Marra M, Bariani R, Mattesi G, Vio R, Bettella N, M DEL, Motta R, Bauce B, Zorzi A, et al. Differential diagnosis of arrhythmogenic cardiomyopathy: phenocopies versus disease variants. *Minerva Med*. 2021;112:269-280. doi: 10.23736/S0026-4806.20.06782-8  87. Karmouch J, Protonotarios A, Syrris P. Genetic basis of arrhythmogenic cardiomyopathy. *Curr Opin Cardiol*. 2018;33:276-281. doi: 10.1097/HCO.0000000000000509  88. Poloni G, De Bortoli M, Calore M, Rampazzo A, Lorenzon A. Arrhythmogenic right-ventricular cardiomyopathy: molecular genetics into clinical practice in the era of next generation sequencing. *J Cardiovasc Med (Hagerstown)*. 2016;17:399-407. doi: 10.2459/JCM.0000000000000385  89. Wicks EC, Elliott PM. Application of current diagnostic criteria for arrhythmogenic right ventricular cardiomyopathy in every day clinical practice. *Curr Pharm Des*. 2015;21:515-524. doi: 10.2174/138161282104141204144402  90. Levanovich PE, Diaczok A, Rossi NF. Clinical and Molecular Perspectives of Monogenic Hypertension. *Curr Hypertens Rev*. 2020;16:91-107. doi: 10.2174/1573402115666190409115330  91. Padmanabhan S, Aman A, Dominiczak AF. Genomics of hypertension. *Pharmacol Res*. 2017;121:219-229. doi: 10.1016/j.phrs.2017.04.031  92. Padmanabhan S, Caulfield M, Dominiczak AF. Genetic and molecular aspects of hypertension. *Circ Res*. 2015;116:937-959. doi: 10.1161/CIRCRESAHA.116.303647  93. James CA, Jongbloed JDH, Hershberger RE, Morales A, Judge DP, Syrris P, Pilichou K, Domingo AM, Murray B, Cadrin-Tourigny J, et al. International Evidence Based Reappraisal of Genes Associated With Arrhythmogenic Right Ventricular Cardiomyopathy Using the Clinical Genome Resource Framework. *Circ Genom Precis Med*. 2021;14:e003273. doi: 10.1161/CIRCGEN.120.003273  94. Oomen A, Semsarian C, Puranik R, Sy RW. Diagnosis of Arrhythmogenic Right Ventricular Cardiomyopathy: Progress and Pitfalls. *Heart Lung Circ*. 2018;27:1310-1317. doi: 10.1016/j.hlc.2018.03.023  95. Castanos Gutierrez SL, Kamel IR, Zimmerman SL. Current Concepts on Diagnosis and Prognosis of Arrhythmogenic Right Ventricular Cardiomyopathy/Dysplasia. *J Thorac Imaging*. 2016;31:324-335. doi: 10.1097/RTI.0000000000000171  96. Iacocca MA, Chora JR, Carrie A, Freiberger T, Leigh SE, Defesche JC, Kurtz CL, DiStefano MT, Santos RD, Humphries SE, et al. ClinVar database of global familial hypercholesterolemia-associated DNA variants. *Hum Mutat*. 2018;39:1631-1640. doi: 10.1002/humu.23634  97. Genomics England PanelApp. Familial hypercholesterolaemia - targeted panel (v. 1.9) [Internet]. 2021 [accessed November 18, 2021]. Available from: https://panelapp.genomicsengland.co.uk/panels/772/.  98. Teekakirikul P, Zhu W, Huang HC, Fung E. Hypertrophic Cardiomyopathy: An Overview of Genetics and Management. *Biomolecules*. 2019;9. doi: 10.3390/biom9120878  99. Pierpont ME, Digilio MC. Cardiovascular disease in Noonan syndrome. *Curr Opin Pediatr*. 2018;30:601-608. doi: 10.1097/MOP.0000000000000669  100. Calcagni G, Adorisio R, Martinelli S, Grutter G, Baban A, Versacci P, Digilio MC, Drago F, Gelb BD, Tartaglia M, et al. Clinical Presentation and Natural History of Hypertrophic Cardiomyopathy in RASopathies. *Heart Fail Clin*. 2018;14:225-235. doi: 10.1016/j.hfc.2017.12.005  101. Bayona-Bafaluy MP, Iglesias E, Lopez-Gallardo E, Emperador S, Pacheu-Grau D, Labarta L, Montoya J, Ruiz-Pesini E. Genetic aspects of the oxidative phosphorylation dysfunction in dilated cardiomyopathy. *Mutat Res Rev Mutat Res*. 2020;786:108334. doi: 10.1016/j.mrrev.2020.108334  102. Padmanabhan S, Dominiczak AF. Genomics of hypertension: the road to precision medicine. *Nat Rev Cardiol*. 2021;18:235-250. doi: 10.1038/s41569-020-00466-4  103. Lip S, Padmanabhan S. Genomics of Blood Pressure and Hypertension: Extending the Mosaic Theory Toward Stratification. *Can J Cardiol*. 2020;36:694-705. doi: 10.1016/j.cjca.2020.03.001  104. Cornu E, Belmihoub I, Burnichon N, Grataloup C, Zinzindohoue F, Baron S, Billaud E, Azizi M, Gimenez-Roqueplo AP, Amar L. [Phaeochromocytoma and paraganglioma]. *Rev Med Interne*. 2019;40:733-741. doi: 10.1016/j.revmed.2019.07.008  105. Burrello J, Monticone S, Buffolo F, Tetti M, Veglio F, Williams TA, Mulatero P. Is There a Role for Genomics in the Management of Hypertension? *Int J Mol Sci*. 2017;18. doi: 10.3390/ijms18061131  106. Louca P, Menni C, Padmanabhan S. Genomic Determinants of Hypertension With a Focus on Metabolomics and the Gut Microbiome. *Am J Hypertens*. 2020;33:473-481. doi: 10.1093/ajh/hpaa022  107. Seidel E, Schewe J, Scholl UI. Genetic causes of primary aldosteronism. *Exp Mol Med*. 2019;51:1-12. doi: 10.1038/s12276-019-0337-9  108. Seidel E, Scholl UI. Genetic mechanisms of human hypertension and their implications for blood pressure physiology. *Physiol Genomics*. 2017;49:630-652. doi: 10.1152/physiolgenomics.00032.2017  109. Geller DS. New Developments in the Genetics of Hypertension: What Should Clinicians Know? *Curr Cardiol Rep*. 2015;17:122. doi: 10.1007/s11886-015-0664-y  110. Precone V, Krasi G, Guerri G, Stuppia L, Romeo F, Perrone M, Marinelli C, Zulian A, Dallavilla T, Bertelli M. Monogenic hypertension. *Acta Biomed*. 2019;90:50-52. doi: 10.23750/abm.v90i10-S.8759  111. Aggarwal A, Rodriguez-Buritica D. Monogenic Hypertension in Children: A Review With Emphasis on Genetics. *Adv Chronic Kidney Dis*. 2017;24:372-379. doi: 10.1053/j.ackd.2017.09.006  112. Zennaro MC, Boulkroun S, Fernandes-Rosa F. Inherited forms of mineralocorticoid hypertension. *Best Pract Res Clin Endocrinol Metab*. 2015;29:633-645. doi: 10.1016/j.beem.2015.04.010  113. Dodoo SN, Benjamin IJ. Genomic Approaches to Hypertension. *Cardiol Clin*. 2017;35:185-196. doi: 10.1016/j.ccl.2016.12.001  114. Luft FC. What Have We Learned from the Genetics of Hypertension? *Med Clin North Am*. 2017;101:195-206. doi: 10.1016/j.mcna.2016.08.015  115. Singh M, Singh AK, Pandey P, Chandra S, Singh KA, Gambhir IS. Molecular genetics of essential hypertension. *Clin Exp Hypertens*. 2016;38:268-277. doi: 10.3109/10641963.2015.1116543 |
| --- |

| **Table S2.** Restrictive panel consisting of genes curated as moderate or higher level of evidence for pathogenicity by ClinGen | | |
| --- | --- | --- |
| **Name** | **Phenotype** | **Highest level of evidence** |
| *ACTC1* | DCM, HCM | Definitive |
| *ACTN2* | DCM, HCM | Moderate |
| *ALPK3* | HCM | Strong |
| *BAG3* | DCM, HCM | Definitive |
| *CACNA1C* | HCM | Definitive |
| *CSRP3* | HCM | Moderate |
| *DES* | DCM, HCM, ARVC | Definitive |
| *DSC2* | ARVC | Definitive |
| *DSG2* | ARVC | Definitive |
| *DSP* | DCM, ARVC | Definitive |
| *FHL1* | HCM | Definitive |
| *FLNC* | DCM, HCM | Definitive |
| *GLA* | HCM | Definitive |
| *JPH2* | DCM, HCM | Moderate |
| *JUP* | ARVC | Definitive |
| *LAMP2* | HCM | Definitive |
| *LMNA* | DCM | Definitive |
| *MYBPC3* | HCM | Definitive |
| *MYH7* | DCM, HCM | Definitive |
| *MYL2* | HCM | Definitive |
| *MYL3* | HCM | Definitive |
| *NEXN* | DCM | Moderate |
| *PKP2* | ARVC | Definitive |
| *PLN* | DCM, HCM, ARVC | Definitive |
| *PRKAG2* | HCM | Definitive |
| *PTPN11* | HCM | Definitive |
| *RAF1* | HCM | Definitive |
| *RBM20* | DCM | Definitive |
| *RIT1* | HCM | Definitive |
| *SCN5A* | DCM | Definitive |
| *TMEM43* | ARVC | Definitive |
| *TNNC1* | DCM, HCM | Definitive |
| *TNNI3* | DCM, HCM | Definitive |
| *TNNT2* | DCM, HCM | Definitive |
| *TPM1* | DCM, HCM | Definitive |
| *TTN* | DCM | Definitive |
| *TTR* | HCM | Definitive |
| *VCL* | DCM | Moderate |

| **Table S3**. Baseline characteristics of the early onset advanced heart failure cohort (SwedeHeartSeq) and the population-based Malmö Diet and Cancer study. | | |
| --- | --- | --- |
|  | **No. (%)** |  |
| **Variable** | **SwedeHeartSeq (n=101)** | **Malmö Diet and Cancer (n=30,447)** |
| Age, mean (SD), y | 50.1 (14.3) | 58.0 (7.6) |
| Sex |  |  |
| Female | 26 (26) | 18,326 (60) |
| Male | 75 (74) | 12,121 (40) |
| Hypertension | 17 (17) | 4,960 (19) |
| Diabetes | 11 (11) | 877 (3) |
| Stroke | 14 (14) | 408 (2) |
| Coronary disease | 18 (18) | 600 (2) |
| CABG | 3 (3) | NA |
| PCI | 15 (15) | NA |
| Smoking |  |  |
| Current | 0 (0.0) | 8087 (28) |
| Previous | 37 (37) | 9658 (34) |
| Never | 64 (63) | 10819 (38) |
| HTx or LVAD |  |  |
| HTx | 97 (96) | NA |
| LVAD | 4 (4) | NA |
| CABG, coronary artery bypass graft; HTx, heart transplant; LVAD, Left ventricular assist device; NA, not available/applicable; PCI, percutaneous coronary intervention. A total of 87 individuals (0.3%) from the MDCS had been diagnosed with HF prior to study inclusion. | | |

| **Table S4.** List of all variants classified as pathogenic, likely pathogenic, or as variants of uncertain significance with suggestive evidence of pathogenicity. Note that a few individuals had more than one variant simultaneously. | | | | | | | |
| --- | --- | --- | --- | --- | --- | --- | --- |
| **Subject** | **Gene** | **HGVSc** | **HGVSp** | **Path. score** | **Class** | **Phenotype** | **Comment** |
| 1 | *TNNT2* | NM_001276345.2:c.776A>C | p.Asp259Ala | 24 | VUS | DCM |  |
| 2 | *PKD1* | NM_001009944.3:c.8299C>T | p.Arg2767Cys | 30 | LP | ICM | Known polycystic kidney disease |
| 3 | *TNNT2* | NM_001276345.2:c.547C>T | p.Arg183Trp | 32 | P | DCM |  |
| 4 | *FLNC* | NM_001458.5:c.4181A>G | p.Lys1394Arg | 30 | VUS | DCM |  |
| 5 | *LAMP2* | NM_002294.3:c.795C>A | p.Cys265Ter | 28 | P | HCM* | Diagnosed with Danon disease. |
| 6 | *TNNT2* | NM_001276345.2:c.547C>G | p.Arg183Gly | 27 | LP | DCM |  |
| 6 | *MYL3* | NM_000258.3:c.170C>G | p.Ala57Gly | 24 | LP | DCM | Low penetrance risk factor. |
| 7 | *MYLK3* | NM_182493.3:c.618dup | p.Ile207HisfsTer9 | 17 | LP | DCM |  |
| 8 | *TTN* | NM_001267550.2:c.13522del | p.Asp4508IlefsTer22 | 19 | LP | DCM | I-band, exon 49 (LRG), PSI 100% |
| 9 | *TTR* | NM_000371.4:c.323A>G | p.His108Arg | 33 | LP | ATTR-CM |  |
| 10 | *TTR* | NM_000371.4:c.220_221delinsTT | p.Glu74Leu | 26 | P | ATTR-CM |  |
| 10 | *POLG* | NM_002693.3:c.1760C>T | p.Pro587Leu | 26 | P | ATTR-CM | Low penetrance risk factor. Interpreted as incidental finding. |
| 10 | *POLG* | NM_002693.3:c.752C>T | p.Thr251Ile | 19 | P | ATTR-CM | Low penetrance risk factor. Interpreted as incidental finding. |
| 11 | *FLNC* | NM_001458.5:c.7813G>A | p.Glu2605Lys | 15 | VUS | HCM |  |
| 12 | *GYG1* | NM_004130.4:c.248C>T | p.Thr83Met | 26 | P | DCM | Diagnosed with GSD XV. |
| 12 | *GYG1* | NM_004130.4:c.487del | p.Asp163ThrfsTer5 | 18 | P | DCM | Diagnosed with GSD XV. |
| 13 | *TTR* | NM_000371.4:c.424G>A | p.Val142Ile | 26 | P | ATTR-CM |  |
| 14 | *TNNI3* | NM_000363.5:c.337G>A | p.Asp113Asn | 24 | VUS | DCM |  |
| 15 | *PLN* | NM_002667.5:c.25C>T | p.Arg9Cys | 32 | P | DCM |  |
| 16 | *GBE1* | NM_000158.4:c.1571G>A | p.Arg524Gln | 31 | LP | HCM* | Diagnosed with GSD IV. |
| 16 | *GBE1* | NM_000158.4:c.1301G>A | p.Arg434Gln | 25 | VUS | HCM* | Diagnosed with GSD IV. |
| 17 | *TNNI3K* | NM_015978.3:c.827+1G>T |  | 26 | VUS | DCM | Predicted to disrupt splicing. |
| 18 | *DSC2* | NM_024422.6:c.1034T>C | p.Ile345Thr | 22 | VUS | ARVC | Homozygous. |
| 19 | *DES* | NM_001927.4:c.1234G>A | p.Glu412Lys | 29 | VUS | ARVC |  |
| 20 | *MYBPC3* | NM_000256.3:c.2490dup | p.His831SerfsTer2 | 24 | P | HCM |  |
| 21 | *MYH7* | NM_000257.4:c.428G>A | p.Arg143Gln | 34 | LP | HCM |  |
| 22 | *TPM1* | NM_001018005.2:c.574G>A | p.Glu192Lys | 29 | P | HCM |  |
| 23 | *LMNA* | NM_170707.4:c.1129C>T | p.Arg377Cys | 37 | P | DCM |  |
| 24 | *MYH7* | NM_000257.4:c.2156G>A | p.Arg719Gln | 29 | P | HCM |  |
| 25 | *TTR* | NM_000371.4:c.323A>G | p.His108Arg | 33 | LP | ATTR-CM |  |
| 26 | *TTN* | NM_001267550.2:c.100295del | p.Arg33432LeufsTer11 | 20 | LP | DCM | A-band, exon 358 (LRG), PSI 100% |
| 27 | *DSG2* | NM_001943.5:c.2315T>G | p.Leu772Ter | 29 | LP | ARVC |  |
| 27 | *DSG2* | NM_001943.5:c.1003A>G | p.Thr335Ala | 19 | VUS | ARVC |  |
| 28 | *LMNA* | NM_170707.4:c.1072_1077del | p.Glu358_Tyr359del | 19 | VUS | DCM |  |
| 29 | *TTN* | NM_001267550.2:c.658C>T | p.Arg220Ter | 22 | LP | ICM | Z-disc, exon 5 (LRG), PSI 100% |
| 30 | *TTN* | NM_001267550.2:c.90566dup | p.Leu30189PhefsTer4 | 20 | LP | DCM | A-band, exon 336 (LRG), PSI 100% |
| 31 | *DMD* | NM_004006.3:c.31+1del |  | 24 | LP | DCM | Predicted to disrupt splicing. Hemizygous. |
| 32 | *PKP2* | NM_001005242.3:c.2014-1G>C |  | 33 | P | ARVC | Predicted to disrupt splicing. |
| 33 | *MYBPC3* | NM_000256.3:c.2490dup | p.His831SerfsTer2 | 24 | P | DCM |  |
| 34 | *LMNA* | NM_170707.4:c.1304_1307dup | p.Ser437HisfsTer2 | 29 | P | DCM |  |
| 35 | *FLNC* | NM_001458.5:c.6826G>A | p.Val2276Met | 26 | VUS | HCM |  |
| 36 | *LMNA* | NM_170707.4:c.992G>A | p.Arg331Gln | 29 | P | ACHD |  |
| 37 | *DES* | NM_001927.4:c.735G>A | p.Glu245%3D | 21 | LP | DCM |  |
| 38 | *ACTC1* | NM_005159.5:c.301G>A | p.Glu101Lys | 31 | P | DCM |  |
| 39 | *MYBPC3* | NM_000256.3:c.2490dup | p.His831SerfsTer2 | 24 | P | DCM |  |
| 39 | *MYBPC3* | NM_000256.3:c.442G>A | p.Gly148Arg | 20 | LP | DCM | Low penetrance risk factor. |
| 40 | *LMNA* | NM_170707.4:c.1304_1307dup | p.Ser437HisfsTer2 | 29 | P | DCM |  |
| 41 | *KCNQ1* | NM_000218.3:c.1588C>T | p.Gln530Ter | 33 | (P) | Myocarditis | Interpreted as secondary finding. |
| 42 | *ACTC1* | NM_005159.5:c.1132T>C | p.Ter378GlnextTer46 | 20 | VUS | DCM |  |
| 43 | *MYLK3* | NM_182493.3:c.1569-2A>C |  | 27 | LP | DCM | Predicted to disrupt splicing. |
| 44 | *DMD* | NM_004006.3:c.5632C>T | p.Gln1878Ter | 30 | P | DCM | Hemizygous. |
| 45 | *KCNQ1* | NM_000218.3:c.1552C>T | p.Arg518Ter | 24 | (P) | HCM | Interpreted as secondary finding. |
| 45 | *MYH7* | NM_000257.4:c.3301G>A | p.Gly1101Ser | 18 | VUS | HCM |  |
| 46 | *TTN* | NM_001267550.2:c.85150C>T | p.Arg28384Ter | 29 | LP | DCM | A-band, exon 327 (LRG), PSI 100% |

All variants were heterozygous unless otherwise stated.

Abbreviations: HGVSc, Human Genome Variation Society [HGVS] coding sequence name; HGVSp, HGVS protein sequence name; Path. score, pathogenicity score as created from prioritization algorithm; ACMG criteria, American College of Medical Genetics criteria^8^; VUS, variant of uncertain significance; LP, likely pathogenic; P, pathogenic; (P), pathogenic but interpreted as secondary finding; DCM, dilated cardiomyopathy (CM); ICM, ischemic cardiomyopathy; HCM, hypertrophic CM; HCM*, HCM phenocopy; ATTR-CM, transthyretin amyloid CM (counted as HCM phenocopy here); ARVC, arrhythmogenic right ventricular CM; ACHD, adult congenital heart disease; LRG, Locus Reference Genomic sequence; PSI, proportion spliced-in^31^; GSD, glycogen storage disease

# **Supplementary Figures**

**Figure S1**. **Genes associated with monogenic heart muscle disease from systematic literature review.** Genes were grouped by cellular organelle of principal function. Refences for the genes are listed in **Supplementary** **Table S1**.


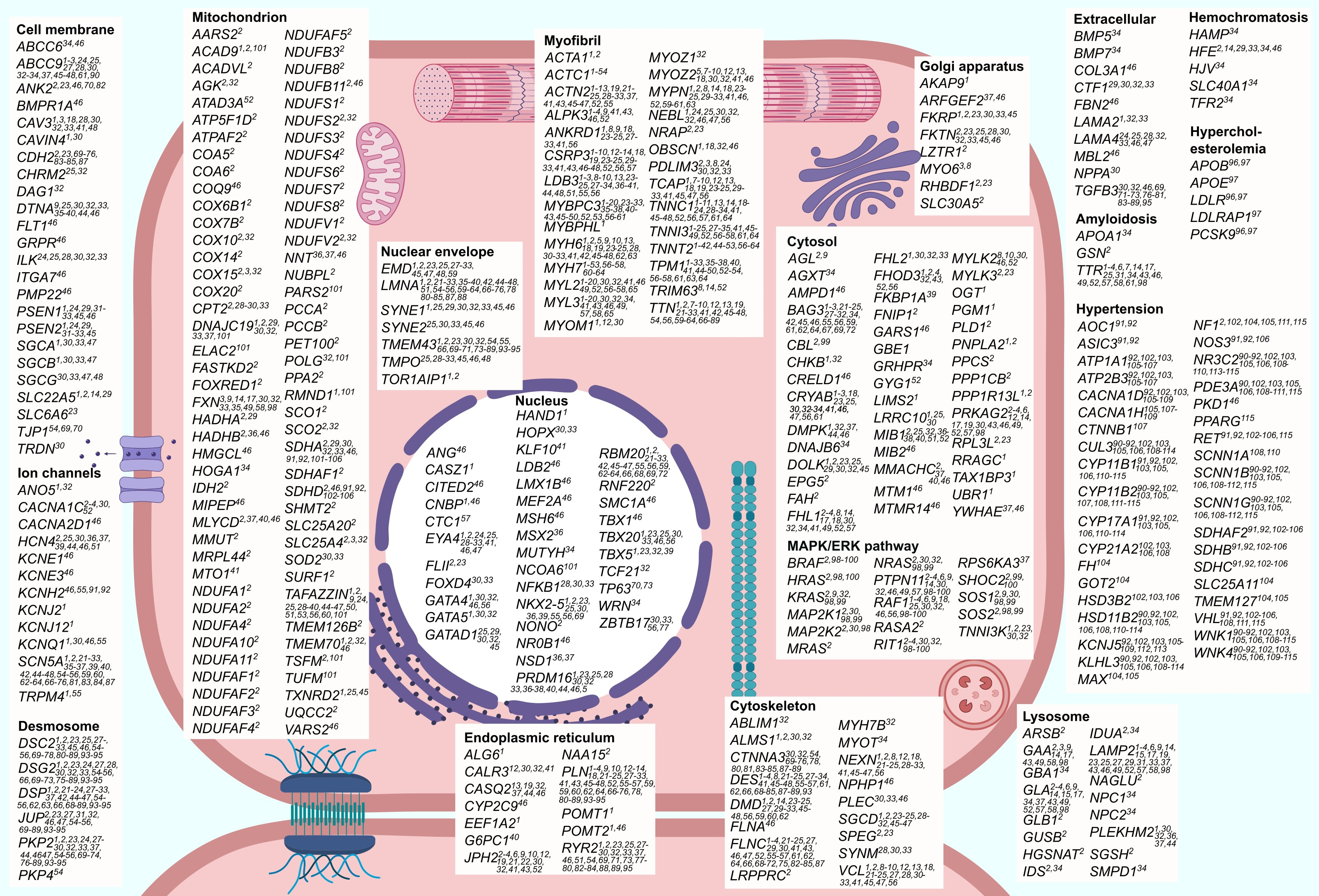


**Figure S2**. **Distribution of polygenic risk scores between heart transplanted individuals with and without an identified likely pathogenic or pathogenic variant.** Scores are standardized to the scores of the population-based Malmö Diet and Cancer cohort.


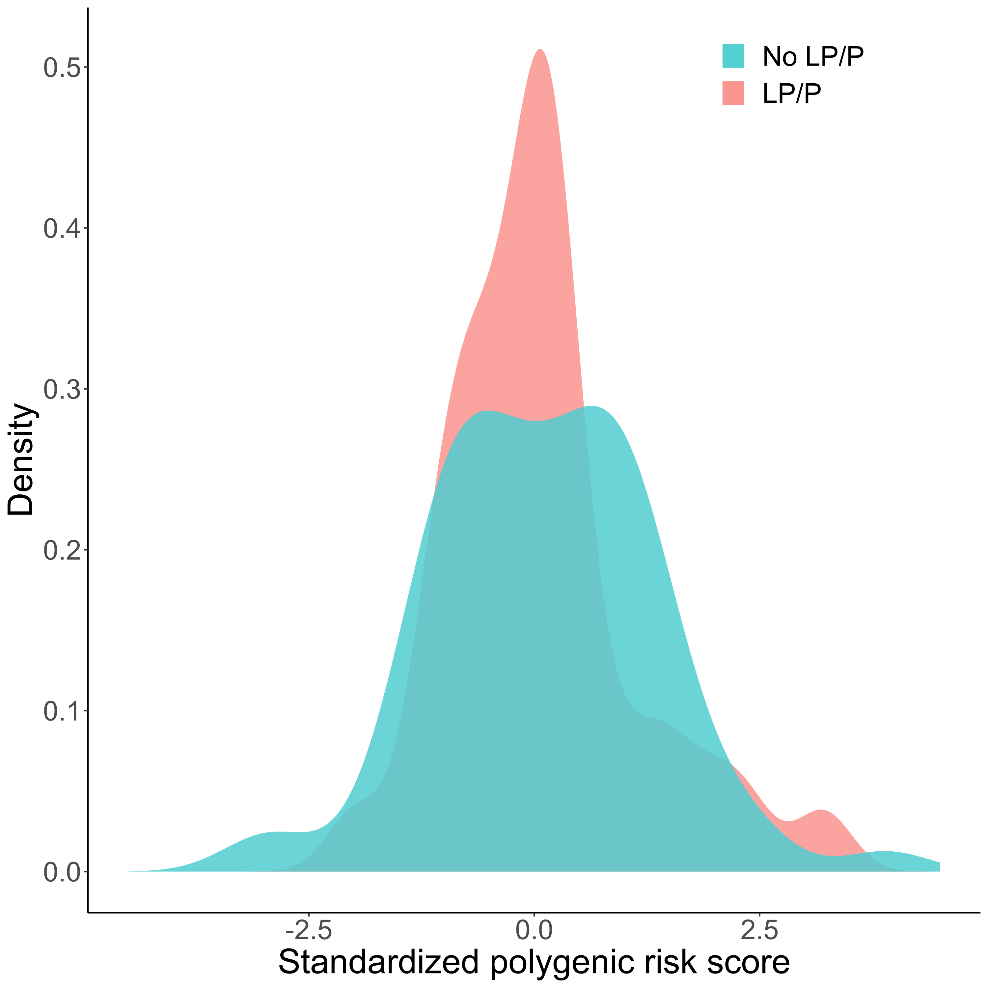

Supplement: Supplementary file 1 — Supplementary Information. [file 41598_2025_88465_MOESM1_ESM.docx]
